# Characterising the background incidence rates of adverse events of special interest for covid-19 vaccines in eight countries: multinational network cohort study

**DOI:** 10.1136/bmj.n1435

**Published:** 2021-06-14

**Authors:** Xintong Li, Anna Ostropolets, Rupa Makadia, Azza Shoaibi, Gowtham Rao, Anthony G Sena, Eugenia Martinez-Hernandez, Antonella Delmestri, Katia Verhamme, Peter R Rijnbeek, Talita Duarte-Salles, Marc A Suchard, Patrick B Ryan, George Hripcsak, Daniel Prieto-Alhambra

**Affiliations:** 1 Centre for Statistics in Medicine, NDORMS, University of Oxford, Oxford, UK; 2 Department of Biomedical Informatics, Columbia University Irving Medical Center, New York, NY, USA; 3 Janssen Research and Development, Titusville, NJ, USA; 4 Neurology Department, Hospital Clinic de Barcelona and University of Barcelona, Barcelona, Spain; 5 Fundacio Institut Universitari per a la recerca a l’Atencio Primaria de Salut Jordi Gol i Gurina (IDIAPJGol), Barcelona, Spain; 6 Department of Medical Informatics, Erasmus University Medical Center, Rotterdam, Netherlands; 7 Department of Bio-Analysis, Faculty of Pharmaceutical Sciences, Ghent University, Ottergemsesteenweg, Gent, Belgium; 8 Department of Biostatistics, Fielding School of Public Health, University of California, Los Angeles, Los Angeles, CA, USA; 9 Department of Human Genetics, David Geffen School of Medicine at UCLA, University of California, Los Angeles, Los Angeles, CA, USA

## Abstract

**Objective:**

To quantify the background incidence rates of 15 prespecified adverse events of special interest (AESIs) associated with covid-19 vaccines.

**Design:**

Multinational network cohort study.

**Setting:**

Electronic health records and health claims data from eight countries: Australia, France, Germany, Japan, the Netherlands, Spain, the United Kingdom, and the United States, mapped to a common data model.

**Participants:**

126 661 070 people observed for at least 365 days before 1 January 2017, 2018, or 2019 from 13 databases.

**Main outcome measures:**

Events of interests were 15 prespecified AESIs (non-haemorrhagic and haemorrhagic stroke, acute myocardial infarction, deep vein thrombosis, pulmonary embolism, anaphylaxis, Bell’s palsy, myocarditis or pericarditis, narcolepsy, appendicitis, immune thrombocytopenia, disseminated intravascular coagulation, encephalomyelitis (including acute disseminated encephalomyelitis), Guillain-Barré syndrome, and transverse myelitis). Incidence rates of AESIs were stratified by age, sex, and database. Rates were pooled across databases using random effects meta-analyses and classified according to the frequency categories of the Council for International Organizations of Medical Sciences.

**Results:**

Background rates varied greatly between databases. Deep vein thrombosis ranged from 387 (95% confidence interval 370 to 404) per 100 000 person years in UK CPRD GOLD data to 1443 (1416 to 1470) per 100 000 person years in US IBM MarketScan Multi-State Medicaid data among women aged 65 to 74 years. Some AESIs increased with age. For example, myocardial infarction rates in men increased from 28 (27 to 29) per 100 000 person years among those aged 18-34 years to 1400 (1374 to 1427) per 100 000 person years in those older than 85 years in US Optum electronic health record data. Other AESIs were more common in young people. For example, rates of anaphylaxis among boys and men were 78 (75 to 80) per 100 000 person years in those aged 6-17 years and 8 (6 to 10) per 100 000 person years in those older than 85 years in Optum electronic health record data. Meta-analytic estimates of AESI rates were classified according to age and sex.

**Conclusion:**

This study found large variations in the observed rates of AESIs by age group and sex, showing the need for stratification or standardisation before using background rates for safety surveillance. Considerable population level heterogeneity in AESI rates was found between databases.

## Introduction

On 11 March 2020, the World Health Organization declared the outbreak of covid-19, caused by the SARS-CoV-2 virus, a global pandemic. As of March 2021, more than 100 million confirmed cases and 2.7 million deaths have been reported worldwide.^1^ Vaccines for covid-19 have been developed at unprecedented speed, with phase III clinical efficacy trials reporting results for some vaccines less than a year after WHO declared the pandemic. Since December 2020, several vaccines have been authorised by regulators such as the European Medicines Agency, the US Food and Drug Administration, and the UK Medicines and Healthcare products Regulatory Agency. Large scale immunisation programmes are ongoing worldwide.

Although the speed of vaccine development should be acknowledged, most vaccines have received approval for emergency use only, based on limited trial data. Uncertainty remains about the safety and effectiveness of these vaccines in populations other than those analysed in trials. As with all medicinal products, vaccine safety must continue to be monitored after regulatory authorisation to complement what was learnt during clinical development. Spontaneous adverse event reporting is a foundational component of post-approval pharmacovigilance activities to ensure the safe and appropriate use of medicinal products. Observational healthcare data captured during routine clinical care, such as electronic health records and administrative claims, can augment pharmacovigilance by providing real world information about potential adverse events and the rates of such events in populations of interest. Background rates of adverse events have historically played an important role in monitoring the safety of vaccines by serving as a baseline comparator for observed rates among those vaccinated.^2^ ^3^ Each new vaccine has potential adverse events of special interest (AESIs) that warrant focused evaluation, based on what is known about previous vaccines and a vaccine’s development.

Regulatory agencies around the world have been preparing safety surveillance strategies for covid-19 vaccines. The FDA Center for Biologics Evaluation and Research published a protocol on background AESI rates to monitor the safety of covid-19 vaccines.^4^ The vACCine covid-19 monitoring readinESS (ACCESS) project funded by the EMA also included estimation of background AESI rates in its protocol.^5^ The WHO Council for International Organizations of Medical Sciences recommends using a local population’s own data when defining background rates of AESIs for comparison.^6^ The Observational Health Data Sciences and Informatics community therefore collaborated to design and execute an international open science study to characterise the background rates of AESIs potentially associated with covid-19 vaccines. We carried out this population based network retrospective cohort study, using observational data from 13 databases in eight countries—Australia, France, Germany, Japan, the Netherlands, Spain, the UK, and the US—to describe, in an epidemiological context, AESIs potentially associated with covid-19 vaccines.

## Methods

### Data sources

Data were obtained from 13 databases—eight comprising electronic health records and five comprising administrative claims.

The electronic health record databases were: IQVIA Australia Electronic Medical Records (IQVIA_AUSTRALIA); Integrated Primary Care Information (IPCI_NETHERLANDS), a primary care records database from the Netherlands^7^; IQVIA Longitudinal Patient Data France (IQVIA_FRANCE)^8^; IQVIA Disease Analyser Germany (IQVIA_GERMANY); Information System for Research in Primary Care (SIDIAP_H_SPAIN),^9^ a primary care records database that covers more than 80% of the population of Catalonia, Spain; Clinical Practice Research Datalink (CPRD_GOLD_UK), which consists of data collected from UK primary care for all ages^10^; Columbia University Irving Medical Center (CUMC_US), which covers more than 4.5 million people treated at the New York-Presbyterian Hospital/Columbia University Irving Medical Center in the US; and Optum De-Identified Electronic Health Record Dataset (OPTUM_EHR_US), which covers more than 103 million patients and more than 7000 hospitals and clinics across the US.^11^

The claims based databases were the Japan Medical Data Center (JMDC_JAPAN)^12^ and four US administrative claims databases: IBM MarketScan Commercial Claims and Encounters Database (CCAE_US),^13^ IBM MarketScan Medicare Supplemental and Coordination of Benefits Database (MDCR_US), IBM MarketScan Multi-State Medicaid Database (MDCD_US), and Optum De-Identified Clinformatics Data Mart Database–Socio-Economic Status (OPTUM_SES_US).^11^

The CPRD-GOLD (UK), IQVIA (France, Germany, and Australia), and IPCI (the Netherlands) databases included primary care data, not information on hospital admissions. Regional electronic health records data such as in the CUMC_US might incompletely capture medical events that are recorded in other healthcare institutions. The claims based sources offered relatively complete data on inpatient, outpatient, and prescriptions and treatment, but lacked measurement data and laboratory results (see table 1 and appendix table 1 for detailed descriptions of the databases).

**Table 1:** Characteristics of included populations, stratified by database. Values are numbers (percentages) unless stated otherwise

	CCAE_US	MDCD_US	MDCR_US	OPTUM_EHR_US	OPTUM_SES_US	CUMC_US	CPRD_GOLD_UK	IPCI_NETHERLANDS	SIDIAP_H_SPAIN	IQVIA_FRANCE	IQVIA_GERMANY	IQVIA_AUSTRALIA	JMDC_JAPAN
Full name	IBM MarketScan Commercial claims and encounters Database	IBM MarketScan Multi-State Medicaid Database	IBM MarketScan Medicare Supplemental and Coordination of Benefits Database	Optum Dei-Identified Electronic Health Record Data	Optum De-Identified Clinformatics Data Mart Database–Socio-Economic Status	Columbia University Irving Medical Center	Clinical Practice Research Datalink	Integrated Primary Care Information	Information System for Research in Primary Care-Hospitalization Linked Data	IQVIA Longitudinal Patient Data France	IQVIA Disease Analyser Germany	IQVIA Australia Electronic Medical Records	Japan Medical Data Center
Data type	Claims	Claims	Claims	EHR	Claims	EHR	EHR	EHR	EHR	EHR	EHR	EHR	Claims
Country	US	US	US	US	US	US	UK	Netherlands	Spain	France	Germany	Australia	Japan
Total No of patients	25 315 777	12 966 011	1 533 709	40 955 085	18 643 608	1 164 196	4 532 766	1 536 283	2 217 536	1 746 371	9 295 525	252 212	6 501 991
Person years	42 889 550	23 203 712	2 484 782	72 328 897	32 474 685	2 174 312	9 638 136	3 326 570	5 497 613	3 008 350	16 784 613	383 668	12 848 482
Age group (years):													
1-5	1 256 501 (4.96)	1 755 796 (13.54)	0.0	1 852 425 (4.52)	627 032 (3.36)	40 678 (3.49)	245 525 (5.42)	78 848 (5.13)	99 838 (4.50)	99 309 (5.69)	308 728 (3.32)	13 430 (5.32)	414 167 (6.37)
6-17	4 122 110 (16.28)	4 188 247 (32.30)	0.0	4 773 000 (11.65)	1 930 638 (10.36)	105 520 (9.06)	635 115 (14.01)	211 037 (13.74)	260 102 (11.73)	268 591 (15.38)	823 235 (8.86)	31 780 (12.60)	1 044 041 (16.06)
18-34	6 395 387 (25.26)	2 885 991 (22.26)	0.0	8 182 549 (19.98)	3 331 356 (17.87)	199 020 (17.10)	946 153 (20.87)	304 971 (19.85)	374 994 (16.91)	328 759 (18.83)	1 411 620 (15.19)	50 995 (20.22)	1 533 866 (23.59)
35-54	8 096 864 (31.98)	2 006 493 (15.48)	0.0	10 737 664 (26.22)	4 389 220 (23.54)	300 818 (25.84)	1 217 618 (26.86)	394 868 (25.70)	663 537 (29.92)	446 804 (25.58)	2 338 535 (25.16)	69 872 (27.70)	2 330 010 (35.84)
55-64	4 716 207 (18.63)	1 004 957 (7.75)	0.0	6 655 199 (16.25)	2 384 571 (12.79)	183 612 (15.77)	594 115 (13.11)	219 990 (14.32)	288 494 (13.01)	229 016 (13.11)	1 580 565 (17.0)	36 329 (14.40)	880 065 (13.54)
65-74	728 708 (2.88)	633 262 (4.88)	733 157 (47.80)	4 829 968 (11.79)	3 106 611 (16.66)	171 940 (14.77)	469 682 (10.36)	180 581 (11.75)	246 763 (11.13)	197 816 (11.33)	1 279 048 (13.76)	27 272 (10.81)	279 277 (4.30)
75-84	0.0	341 267 (2.63)	536 970 (35.01)	2 652 453 (6.48)	1 985 356 (10.65)	110 883 (9.52)	290 225 (6.40)	104 288 (6.79)	180 903 (8.16)	117 067 (6.70)	1 191 402 (12.82)	15 319 (6.07)	20 565 (0.32)
≥85	0.0	149 998 (1.16)	263 582 (17.19)	1 271 827 (3.11)	888 824 (4.77)	51 725 (4.44)	134 333 (2.96)	41 700 (2.71)	102 905 (4.64)	59 009 (3.38)	362 392 (3.90)	7215 (2.86)	0.0
Sex:													
Female	13 037 440 (51.50)	7 322 471 (56.47)	849 301 (55.38)	23 220 748 (56.70)	9 595 675 (51.47)	693 190 (59.54)	2 287 698 (50.47)	783 660 (51.01)	1 120 373 (50.52)	926 180 (53.03)	5 340 273 (57.45)	137 203 (54.40)	2 926 702 (45.01)
Male	12 278 337 (48.50)	5 643 540 (43.53)	684 408 (44.62)	17 734 337 (43.30)	9 047 933 (48.53)	471 006 (40.46)	2 245 068 (49.53)	752 623 (48.99)	1 097 163 (49.48)	820 191 (46.97)	3 955 252 (42.55)	115 009 (45.60)	3 575 289 (54.99)

EHR=electronic health record.

All datasets were previously mapped to the Observational Medical Outcomes Partnership common data model, which is maintained by the Observational Health Data Sciences and Informatics network, an international open science initiative to generate reproducible evidence from observational data.^14^ This initiative brings together hundreds of researchers from 30 countries, working with health records from around 600 million unique patients in its distributed database. The analysis code was distributed across all centres contributing to Observational Health Data Sciences and Informatics without sharing patient level data.^15^ ^16^

### Study participants

The study period was from 1 January 2017 to 31 December 2019. We defined the target at risk population as people who were observed on 1 January 2017, 1 January 2018, or 1 January 2019 and were observed for at least 365 days before this observation date. The 1 January each year was defined as the index date.

### Events of interest

The events of interest in this study were AESIs that might need evaluation after covid-19 vaccination. This list of outcomes was based on the protocol published by the FDA Center for Biologics Evaluation and Research, the prioritised covid-19 vaccine AESI list by the Brighton Collaboration, and previous studies.^4^ ^17^ We included 15 events: non-haemorrhagic and haemorrhagic stroke, acute myocardial infarction, deep vein thrombosis, pulmonary embolism, anaphylaxis, Bell’s palsy, myocarditis or pericarditis, narcolepsy, appendicitis, immune thrombocytopenia, disseminated intravascular coagulation, encephalomyelitis (including acute disseminated encephalomyelitis), Guillain-Barré syndrome, and transverse myelitis.^4^

Events were identified by records of the occurrence of conditions based on predefined phenotyping algorithms (eg, diagnosis codes from claims or diagnosis codes and problem lists from electronic health records). Definitions for encephalomyelitis, non-haemorrhagic and haemorrhagic stroke, and acute myocardial infarction also required the record to occur within an inpatient setting in any diagnosis positions, whereas the definition for Guillain-Barré syndrome required the condition to be recorded in an inpatient setting in the primary position. Appendix tables 2 and 3 present the full specifications of all phenotype definitions, including source codes (original codes used in the database) and standard concepts (normative expressions used to represent a unique clinical entity within the Observational Medical Outcomes Partnership common data model, which were mostly SNOMED (Systematized Nomenclature of Medicine) codes in this study).

We defined a “clean window” period before each index date, during which qualifying events (AESIs) could not be observed. If an AESI was observed during this period, the participant did not enter the study cohort for that event. If an individual had a qualified event during follow-up, this participant would contribute to the person time of that event cohort after the clean window continually until censored from the cohort.

**Fig 1:**
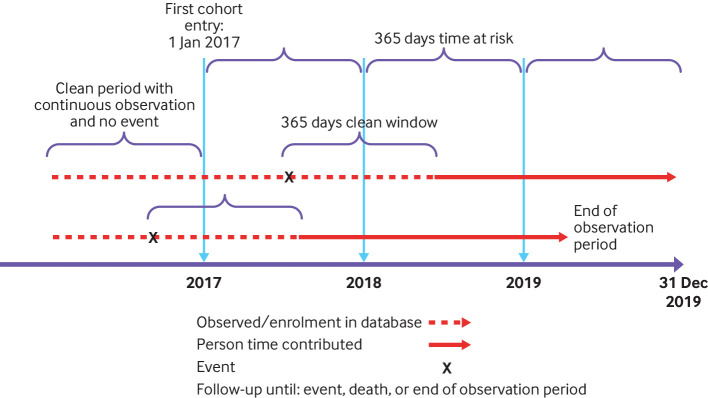
Study design

Figure 1 shows the cohort entry, follow-up, and event definitions. In keeping with the FDA protocol, the clean window was 365 days for all events except anaphylaxis (30 days) and facial nerve palsy and encephalomyelitis (183 days).^4^

As the CPRD-GOLD (UK), IQVIA (France, Germany, and Australia), and IPCI (the Netherlands) databases only included primary care data, we did not use them for events where definition required an inpatient diagnosis.

### Statistical analysis

We defined the time at risk as a 365 day period after the index date. Eligible people contributed time at risk from 1 January to 31 December for each qualifying year in 2017 to 2019. Participants were censored if an event occurred during that event’s clean window, at death (if available in the data source), or at the end of their observation period in the database (fig 1). One participant could contribute more than one event. To avoid duplicate counts we used outcome specific prespecified clean windows of 30 to 365 days.

Incidence rates were estimated as the total number of events divided by the person time at risk per 100 000 person years. We calculated the age and sex specific incidence rates in each database and report all rates when the event counts exceeded a minimum cell count of 5. Age was calculated as year of index date minus year of birth and was partitioned into eight mutually exclusive age groups (in years): 1-5, 6-17, 18-34, 35-45, 55-64, 65-74, 75-84, and 85 and older. Age-sex specific rates of each AESI were pooled across all databases using a random effects meta-analysis, with the DerSimonian-Laird method to estimate variance between databases.^18^ We estimated 95% prediction intervals using the R package “meta”.^19^ The prediction interval reflects the expected uncertainty if an estimate rate from a new study is included in the meta-analysis.^20^

Meta-analytic age and sex specific rates were classified using the WHO Council for International Organizations of Medical Sciences thresholds: very common (≥1/10), common (<1/10 to ≥1/100), uncommon (<1/100 to ≥1/1000), rare (<1/1000 to ≥1/10 000), and very rare (<1/10 000).^21^

All statistical analyses were performed in R software.^22^ The study protocol and analysis code are available at https://github.com/ohdsi-studies/Covid19VaccineAesiIncidenceCharacterization.

### Patient and public involvement

No patients or members of the public were directly involved in the design or analysis of the reported data. Because of covid-19 related restrictions, it has been difficult to interact with relevant patient and public representatives. Some of the contributing databases did, however, involve patients in the evaluation of our data access application.

## Results

From the 13 databases, 126 661 070 people contributed 227 043 370 person years of follow-up. The Optum De-Identified Electronic Health Record Dataset (OPTUM_EHR_US) contributed the largest number of patients (n=40 955 085), followed by the IBM MarketScan Commercial Claims and Encounters Database (CCAE_US). Each database captured important population demographics and collectively represented all age and sex subgroups from eight countries (table 1).

Most of the databases included more female than male patients (ranging from 50.5% female patients in SIDIAP_H_SPAIN to 57.5% in IQVIA_GERMANY), except for JMDC_JAPAN (45.0% female patients). The CCAE_US database included patients aged 0-74 years, whereas the MDCR_US database only included patients older than 65 years. The other databases included patients of all ages. Patients aged 35-54 years accounted for the largest proportion of the population in most databases (from 23.5% in OPEUM_SES_US to 35.8% in JMDC_JAPAN). Patients aged 18-34 years, however, accounted for the largest proportion (22.3%) of the IBM MarketScan Multi-State Medicaid Database (MDCD_US) database. The proportion of patients aged 65 years and older ranged from 32.1% in the OPTUM_SES_US database to less than 10% in the CCAE_US, JMDC_JAPAN, and MDCD_US databases. Patients younger than 18 years accounted for 45.8% of the MDCD_US database.

**Fig 2:**
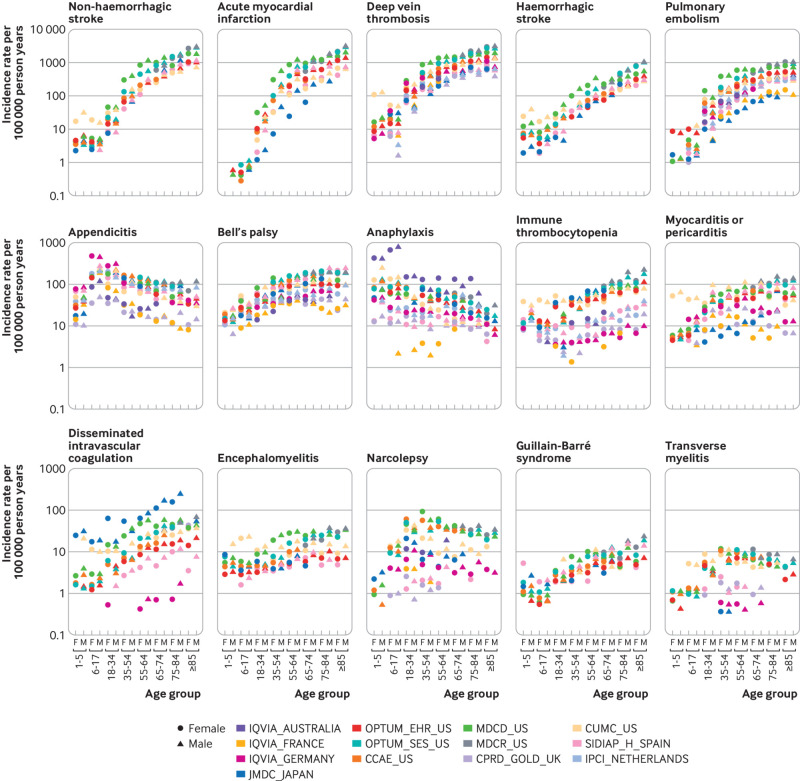
Age and sex stratified incidence rates for 15 adverse events of special interest by database. CCAE_US=IBM MarketScan Commercial Claims and Encounters Database, CPRD_GOLD_UK=Clinical Practice Research Datalink; CUMC_US=Columbia University Irving Medical Center; IPCI_NETHERLANDS=Integrated Primary Care Information; IQVIA_AUSTRALIA=IQVIA Australia Electronic Medical Records; IQVIA_FRANCE=IQVIA Longitudinal Patient Data France; IQVIA_GERMANY=IQVIA Disease Analyser Germany; JMDC_JAPAN=Japan Medical Data Center, MDCD_US=IBM MarketScan Multi-State Medicaid Database, MDCR_US=IBM MarketScan Medicare Supplemental and Coordination of Benefits Database; OPTUM_EHR_US=Optum De-Identified Electronic Health Record Dataset; OPTUM_SES_US=Optum De-Identified Clinformatics Data Mart Database-Socio-Economic Status; SIDIAP_H_SPAIN=Information System for Research in Primary Care-Hospitalization Linked Data

Substantial heterogeneity was observed by age group and sex in the database specific estimates of each event but similar age and sex trends in most databases and the pooled rates (fig 2). For example, the incidence rates of deep vein thrombosis increased with age. In the OPTUM_SES_US data, the incidence rates increased from 20 (95% confidence interval 19 to 22) per 100 000 person years among boys aged 6-17 years to 2030 (2009 to 2051) per 100 000 person years among men aged 75-84 years (see appendix table 4). Similarly, myocardial infarction rates among men increased from 28 (95% confidence interval 27 to 29) per 100 000 person years in those aged 18-34 years to 1400 (1374 to 1427) per 100 000 person years in those older than 85 years in the OPTUM_EHR_US data. The incidence rates for haemorrhagic and non-haemorrhagic stroke, pulmonary embolism, Bell’s palsy, immune thrombocytopenia, Guillain-Barré syndrome, and disseminated intravascular coagulation also increased with age.

The rates of haemorrhagic stroke were higher in male participants than female participants in most age groups. For example, the incidence rates among those aged 65-74 years in the MDCR_US database were 251 (238 to 265) per 100 000 person years for male participants and 170 (160 to 180) per 100 000 person years for female participants. The incidence rates of acute myocardial infraction, myocarditis or pericarditis, immune thrombocytopenia, and Guillain-Barré syndrome were also higher in male participants than female participants.

**Fig 3:**
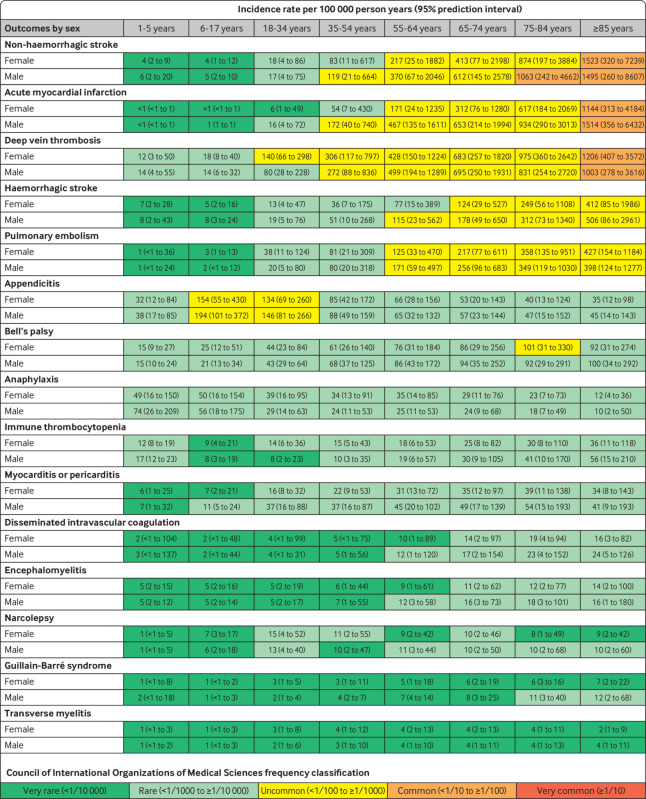
Pooled estimated age and sex stratified incidence rates per 100 000 person years (95% prediction intervals), calculated from meta-analyses

Figure 3 summarises the pooled incidence rates of the 15 AESIs, stratified by age and sex, based on prediction intervals from a meta-analysis of the database estimates. Each age and sex subgroup was classified using the Council for International Organizations of Medical Sciences adverse event frequency system (very common, common, uncommon, rare, or very rare). The AESIs studied spanned the continuum of possible frequencies. The incidence of several events varied substantially by age and was therefore classified differently at different ages. For example, deep vein thrombosis was rare in boy and girl participants younger than 18 years, uncommon in those aged 35-84 years, and common in those 85 years and older. Acute myocardial infarction was very rare (<1/10 000) in women younger than 35 years, rare (<1/1000 to ≥1/10 000) in women aged 35-54 years, uncommon (<1/100 to ≥1/1000) in both men and women aged 55-84 years, and common (<1/10 to ≥1/100) in men and women aged 85 years and older. Anaphylaxis, Bell’s palsy, appendicitis, and immune thrombocytopenia were largely rare in all age groups, although appendicitis was uncommon in those aged 6-34 years. Guillain-Barré syndrome and transverse myelitis were very rare in nearly all subgroups.

The rates recorded for deep vein thrombosis highlight the population level variation. Thirteen database estimates were obtained for the incidence in women aged 65-74 years, ranging from 387 (370 to 404) per 100 000 person years in CPRD-GOLD to 1443 (1416 to 1470) per 100 000 person years in MDCD_US. The rates in eight databases were less than 650 per 100 000 person years (CPRD-GOLD in the UK; CUIMC in the US; IPCI in the Netherlands; IQVIA in Australia, France, and Germany; JMDC in Japan; and SIDIAP-H in Spain), whereas the rates in three databases were more than twice as high, at more than 1300 per 100 000 person years (MDCD, MDCR, and OPTUM-SES in the US). For women aged 35-54 years, the incidence rates ranged from 159 (151 to 167) per 100 000 person years in Spain (SIDIAP) to 866 (854 to 878) per 100 000 person years in the US (MDCD). Among women aged 75-84 years, the lowest incidence rate was 585 (559 to 612) per 100 000 person years (CPRD-GOLD in the UK) and the highest was 2167 (2126 to 2210) per 100 000 person years (MDCR in the US). No consistent patterns could be found to explain why certain databases yielded higher or lower rates across outcomes. Database specific rates are shown in figure 2, and the raw numbers are available in appendix table 4 and our interactive web app (https://data.ohdsi.org/Covid19VaccineAesiIncidenceCharacterization/).

## Discussion

We conducted a multinational network cohort study on the descriptive epidemiology of the AESIs prioritised for post-marketing surveillance of covid-19 vaccines. We report background rates of deep vein thrombosis, pulmonary embolism, stroke, immune thrombocytopenia, and disseminated intravascular coagulation. These events are particularly relevant for covid-19 vaccines as the SARS-COV-2 virus has been observed to affect coagulopathy.^23^ ^24^ ^25^ ^26^ We assessed the incidence rates of 15 AESIs across 13 databases, eight countries, and four continents. We observed considerable variability with age and sex, emphasising the need for standardisation or stratification of the background rates used for vaccine surveillance. We found substantial heterogeneity between databases, suggesting that, where possible, post-covid-19 vaccine rates should be compared with background, or historical, rates obtained from the same dataset.

### Research in context

In vaccine safety surveillance, background incidence rates have been used to estimate the expected number of events in the general population, which is also known as the historical rates comparison method.^3^ ^27^ Several vaccine safety surveillance guidelines, such as the European Network of Centres of Pharmacoepidemiology and Pharmacovigilance, suggest using this historical rate comparison method, as it increases statistical power to detect rare events and helps to detect signals sooner than other methods. This method has been widely implemented in many countries and by many organisations, including the Vaccine Safety Datalink project in the US^28^ and the Vaccine Adverse Event Surveillance and Communication project in Europe.^3^ These background rates are often obtained from the literature or healthcare databases. However, use of historical rate comparisons has some limitations owing to the methods used to obtain the rates, case and population definitions, differences in clinical codes, and geographical and temporal variations.^28^ We overcame these common limitations by calculating the presented estimates using the same setting and common analysis procedures, phenotyping algorithms, and common data model.

We found substantial population level heterogeneity across data sources for all events, even after standardising outcome definitions and stratifying by age and sex. For example, we observed about a threefold difference between the highest and lowest incidence rates for deep vein thrombosis measured in each database. Previous studies using one or a small number of databases have also observed these variations. US based studies, for example, recorded incidence rates per 100 000 person years for idiopathic thrombocytopenia of around 3 among men and women aged 26-62 years,^29^ 9 among those aged 25-44 years, and 12 among those aged 45-64 years.^30^ We estimated incidence rates of transverse myelitis ranging from 1 to 4 per 100 000 person years in meta-analyses, depend on age and sex strata. Previous studies have reported overall incidence rates of transverse myelitis ranging from 0.4 to 4.6 per 100 000 person years.^27^ ^31^

Recorded rates of Bell’s palsy among those older than 65 years have ranged from 4.6 per 100 000 person years in Italian data to 174 per 100 000 person years in US data.^30^ ^32^ We found similar rates to those previously published using US claims databases and UK general practice data, but higher rates when using Spanish data. A previous study using data from regions in Spain not captured by the SIDIAP database found an incidence rate of 63 per 100 000 person years among those older than 65 years.^32^ We observed rates of 131 and 182 per 100 000 person years in women and men aged 65-74 years, respectively, using SIDIAP Spanish data (SIDIAP_H_SPAIN). A US study found rates of narcolepsy between 31 and 38 per 100 000 person years among those aged 25-64 years, whereas studies based on European data have found much lower rates of between 0.2 and 2.5 per 100 000 person years for the same age group.^3^ ^30^ ^32^ We also found higher rates of narcolepsy in US databases than those from other countries. Recently reported data from the ACCESS project also showed heterogeneity in background rates.^5^ This heterogeneity must be considered when comparing rates across populations.

Most of the studied outcomes also had considerable within source patient level heterogeneity that followed age and sex patterns. We observed that the rates of cardiovascular diseases such as acute myocardial infarction, haemorrhagic and non-haemorrhagic stroke, deep vein thrombosis, and pulmonary embolism increased with age. The incidence of Guillain-Barré syndrome and Bell’s palsy also increased with age. Narcolepsy and appendicitis were more common in younger populations. The patterns observed in our study were generally comparable with those of previous reports.^2^ ^3^ ^29^ ^30^ ^32^ ^33^ ^34^ Stratification by age and sex and standardisation are likely to be useful analytical strategies to reduce confounding when incidence rates are compared across populations. The observed magnitude of heterogeneity across sources within age and sex subgroups, however, suggests that residual patient level differences will remain, including differences in the distributions of other risk factors, such as comorbidities and medication use.

Comparing published results can be complicated by differing study methods, including the time-at-risk definition, study period, event definitions, population coverage, calendar year, and geographical location.^35^ Different subgroup definitions also make direct comparison difficult. Previous studies of Guillain-Barré syndrome, for example, have used age strata that do not fully overlap with each other.^2^ ^5^ ^29^ ^32^ ^36^ ^37^ As we used the same definitions, data model, and analysis with all of our studied databases, the heterogeneity we observed cannot be attributed to variability in analysis. This remaining heterogeneity might have resulted from differences in the underlying populations, healthcare systems, and data capture processes. Although some variability might have been due to systematic error, selection bias, or differential outcome measurement error between databases, some could reflect true population differences, such as socioeconomic status and comorbidities.

As we observed notable differences in incidence rates by age, sex, and database, caution is needed when incidence rates are compared across time or populations. Incidence rates from different sources might be subject to substantial systematic error. Given this heterogeneity, the reported 95% confidence intervals for the database specific rates in our study cannot reflect the systematic errors in the rate estimation. The notably wide prediction intervals for each age and sex subgroup also reflect the substantial population level heterogeneity observed across sources. We observed large variations between electronic health records and claims data sources when using the same analysis and outcome definitions. Variability in rates derived from randomised trials or spontaneous reporting data could be even greater. If observational databases are to be used to inform safety surveillance activities, within database analyses (such as self-controlled case designs or propensity score adjusted comparative cohort designs) may help reduce study bias for any given comparison. Showing consistent effects across databases may further strengthen confidence in results. If observational data are used to derive historical “expected” rates and compared with observed rates of events from another source, then the uncertainty in the background rate must be appropriately integrated to avoid misleading conclusions.

### Strengths of this study

The large number of participating databases, geographical coverage, and sizable study population enabled a comprehensive assessment of background incidence rates of AESIs across different healthcare systems and regions worldwide. This study is an example of the collaborative projects possible within the Observational Health Data Sciences and Informatics network. This initiative stores records using a common data model^38^ and standard vocabularies and develops and uses state-of-the-art methods to draw and validate causal conclusions.^39^ It has generated impactful evidence in many areas, such as hypertension treatment,^40^ ^41^ and was able to pivot quickly to generate policy influencing evidence in covid-19 management.^42^ ^43^ In our study we took advantage of the Observational Medical Outcomes Partnership common data model, which enabled us to use the same study design and analytical code in all databases and to gather results from participating data partners rapidly and without transferring patient level data. All outcome definitions, clinical codes, and phenotype algorithms have been made open source and are available online for review and to maximise reproducibility and reuse. The large scale use of the Observational Medical Outcomes Partnership common data model and open source science strategy has enabled us to generate useful, timely evidence on upcoming covid-19 vaccine safety. We welcome new data partners to run the analyses on their datasets, contribute their results to our web application, and participate in further Observational Health Data Sciences and Informatics studies.

### Limitations of this study

The primary limitation of this study is that all outcomes could have been subject to measurement error. As the outcome definitions were based on the presence of specific diagnostic codes and were not validated further, sensitivity or specificity could have been imperfect. Our analysis relied on data from 2017 to 2019 using a target population of all people in each database with more than 365 days of observation indexed on 1 January, 365 days’ time at risk, and outcome specific clean windows to allow for recurrent events. The impact of these design decisions should be explored further.

Some limitations relate to the use of each database. As information on hospital admission was not available in the primary care datasets used (CPRD-GOLD in the UK, IQVIA in France, Germany, and Australia, and IPCI in the Netherlands), events that happened during inpatient visits were not included. The electronic health records data sources were subject to incomplete capture of medical events recorded in other healthcare institutions. The bias of incomplete information was partially mitigated by including only those patients who had at least one year of continuous observation. The five administrative claims data sources offered reliable data capture but lacked data elements such as laboratory test results. The US based claims database did not record death information well. Our within database background rate comparison should have minimised bias related to these database specific limitations, mitigating against such limitations.

### Conclusion

Our study assessed the descriptive epidemiology of potential AESIs for covid-19 vaccines. Our study highlights the wide range of adverse effects being monitored, from very rare neurological disorders to more common thromboembolic conditions. We found large variations in the observed rates of AESIs by age group and sex, showing the need for stratification or standardisation before using background rates for safety surveillance. Considerable population level heterogeneity was also found in AESI rates between databases, implying that individual study estimates should be interpreted with caution and that the systematic error associated with database choice should be incorporated into any analysis. We recommend that the same database be used to estimate post-covid-19 vaccine and background rates for comparison in vaccine safety monitoring. The database specific estimates reported here are available in a bespoke interactive web application for regulators and other stakeholders (https://data.ohdsi.org/Covid19VaccineAesiIncidenceCharacterization/). These background rates provide useful real world context to inform public health efforts aimed at ensuring patient safety while promoting the appropriate use of vaccines worldwide.

**What is already known on this topic**

Background rates of adverse events of special interest (AESIs) have historically played an important role in monitoring vaccine safety
Most studies focused on single or very few events and used different study designs, and none focused on specific AESIs for covid-19 vaccines
No international transcontinental study on background rates of covid-19 vaccine AESIs using the same definitions, data model, and analysis across all databases have been reported

**What this study adds**

This study found considerable heterogeneity between geographies and databases, suggesting caution when interpreting the differences between observed and expected rates
If possible, the same data source should be used to compare post-covid-19 vaccine (observed) and background (expected) AESI rates for vaccine surveillance
Considerable variability was also found in observed rates of AESIs between age groups and sex, showing the need for standardisation if background rates are used for surveillance purposes

## Web extra

Extra material supplied by authors

## Notes

Contributors: XL and AO are joint first authors. DAP, XL, PR, and GH conceived the study. XL, AO, GH, PR, and DAP interpreted the results and wrote the manuscript. RM, AZ, GR, and AS implemented the study. XL, AO, PR, RM, KV, PRR, TDS, and MAS contributed to the study execution (data holders). XL, AO, GH, and PR contributed to the study design. All coauthors contributed to writing the manuscript. All authors approved the final version and had final responsibility for the decision to submit for publication. PRR, MAS, PBR, GH, and DPA are guarantors. The corresponding author attests that all listed authors meet authorship criteria and that no others meeting the criteria have been omitted.

Ethical approval: The protocol for this research was approved by the independent scientific advisory committee for Medicine and Healthcare products Regulatory Agency database research (protocol number 20_000211), the IDIAPJGol (Fundacio Institut Universitari per a la recerca a l’Atencio Primaria de Salut Jordi Gol i Gurina) clinical research ethics committee (project code: 21/007-PCV), the Integrated Primary Care Information governance board (application number 3/2021), and Columbia University institutional review board (AAAO7805).

Data sharing: Patient level data cannot be shared without approval from data custodians owing to local information governance and data protection regulations. Aggregated data, analytical code, and detailed definitions of algorithms for identifying the events are available in a GitHub repository (https://github.com/ohdsi-studies/Covid19VaccineAesiIncidenceCharacterization). The results are available in an interactive web app (https://data.ohdsi.org/Covid19VaccineAesiIncidenceCharacterization).

The lead authors (XL and AO) affirm that this manuscript is an honest, accurate, and transparent account of the study being reported; that no important aspects of the study have been omitted; and that any discrepancies from the study as planned (and, if relevant, registered) have been explained.

Dissemination to participants and related patient and public communities: We will disseminate a lay summary of our findings through our Twitter and other social media accounts. The involved universities will lead on the dissemination of our results to the lay audience, with help from our communications and outreach teams in the UK, US, and the Netherlands.

Provenance and peer review: Not commissioned; externally peer reviewed.
