# Supplementary material for: Characterising the background incidence rates of adverse events of special interest for covid-19 vaccines in eight countries: multinational network cohort study
Source: BMJ. 2021 Jun 14;373:n1435. doi: 10.1136/bmj.n1435 (PMC8193077; doi:10.1136/bmj.n1435)

Appendix tables for

Characterizing the background incidence rates of adverse events of special interest for COVID-19 vaccines across eight countries: a multinational network cohort study

AUTHORS: Xintong Li[1]*, Anna Ostropolets[2]*, Rupa Makadia[3], Azza Shoaibi[3], Gowtham Rao[3], Anthony G. Sena[3,6], Eugenia Martinez-Hernandez[4], Antonella Delmestri[1], Katia Verhamme[6], Peter R Rijnbeek[6], Talita Duarte-Salles[5], Marc Suchard[7,8], Patrick B Ryan[2,3], George Hripcsak[2], Daniel Prieto-Alhambra[1,6]
*Joint first authors

AFFILIATIONS:
1. Centre for Statistics in Medicine, NDORMS, University of Oxford, Oxford, United Kingdom
2. Department of Biomedical Informatics, Columbia University Irving Medical Center, New York, NY, USA
3. Janssen Research and Development, Titusville, NJ, USA
4. Neurology Department, Hospital Clinic de Barcelona and University of Barcelona, Barcelona, Spain
5. Fundacio Institut Universitari per a la recerca a l'Atencio Primaria de Salut Jordi Gol i Gurina (IDIAPJGol), Barcelona, Spain
6. Department of Medical Informatics, Erasmus University Medical Center, Rotterdam, The Netherlands
7. Department of Biostatistics, Fielding School of Public Health, University of California, Los Angeles, Los Angeles, CA USA,
8. Department of Human Genetics, David Geffen School of Medicine at UCLA, University of California, Los Angeles, UCLA, Los Angeles, CA, USA

Corresponding author:
Prof Daniel Prieto-Alhambra
Botnar Research Centre, Windmill Road, OX37LD, Oxford, United Kingdom
daniel.prietoalhambra@ndorms.ox.ac.uk

Content

Appendix Table 1: database information
Appendix Table 2: adverse events of special interest phenotype definition and links
Appendix Table 3: source clinical codes used in phenotype definition
Appendix Table 4: age-sex specific crude incidence rates for each events of each database
Appendix Figure 1: database-specific incidence rates and meta-analytic pooled rates with 95% prediction intervals

Appendix Table 1: database information

Data source	Short name	Description
IQVIA Australia Electronic Medical Records (Australia EMR)	**IQVIA_AUSTRALIA**	Anonymized patient records of more than 6 million patients in Australia collected from Patient Management software used by GPs during an office visit to document patients' clinical records.
IQVIA Longitudinal Patient Data France (LPD France)	**IQVIA_FRANCE**	Anonymized patient records of 7.8 million patients in France collected from Patient Management software used by GPs and select specialists during an office visit to document patients' clinical records
IQVIA Disease Analyser Germany (DA Germany)	**IQVIA_GERMANY**	IQVIA DA Germany is collected from extracts of patient management software used by GPs and specialists practicing in ambulatory care settings. Data coverage includes more than 34M distinct person records out of at total population of 80M (42.5%) in the country and collected from 2,734 providers. Dates of service include from 1992 through March 2020.
Japan Medical Data Center (JMDC)	**JMDC_JAPAN**	Japan Medical Data Center (JMDC) database consists of data from 60 society-managed health insurance plans covering workers aged 18 to 65 and their dependents (children younger than 18years old and elderly people older than 65 years old). JMDC data includes membership status of the insured people and claims data provided by insurers under contract (e.g. patient-level demographic information, inpatient and outpatient data inclusive of diagnosis and procedures, and prescriptions as dispensed claims information).
Integrated Primary Care Information (IPCI)	**IPCI_NETHERLANDS**	The Integrated Primary Care Information (IPCI) database is a Dutch database containing the medical records of more than 2.5 million patients provided by more than 600 GPs geographically spread over the Netherlands. In the Netherlands, all citizins are registered with a GP practice which acts as a gatekeeper in a two-way exchange of information with secondary care.
Optum® De-Identified Clinformatics® Data Mart Database – Socio-Economic Status (Optum SES)	**OPTUM_SES_US**	Optum® De-Identified Clinformatics® Data Mart Database is an adjudicated administrative health claims database for members with private health insurance, who are fully insured in commercial plans or Medicare Advantage. The population is primarily representative of US commercial claims patients (0-65 years old) with some Medicare (65+ years old) however ages are capped at 90 years. It includes data captured from administrative claims processed from inpatient and outpatient medical services and prescriptions as dispensed, as well as results for outpatient lab tests processed by large national lab vendors who participate in data exchange with Optum. Optum SES provides socio-economic status for members with both medical and pharmacy coverage and location information for patients at the US Census Division level.

Optum® de-identified Electronic Health Record Dataset (Optum EHR)	**OPTUM_EHR_US**	Optum® de-identified Electronic Health Record Dataset is derived from dozens of healthcare provider organizations in the United States (that include more than 700 hospitals and 7,000 Clinics treating more than 103 million patients) receiving care in the United States. The medical record data includes clinical information, inclusive of prescriptions as prescribed and administered, lab results, vital signs, body measurements, diagnoses, procedures, and information derived from clinical Notes using Natural Language Processing (NLP).
IBM MarketScan Medicare Supplemental and Coordination of Benefits Database (MDCR)	**MDCR_US**	IBM MarketScan Medicare Supplemental and Coordination of Benefits Database (MDCR) representshealth services of retirees in the United States with primary or Medicare supplemental coveragethrough privately insured fee-for-service, point-of-service, or capitated health plans. These datainclude adjudicated health insurance claims (e.g. inpatient, outpatient, and outpatient pharmacy).Additionally, it captures laboratory tests for a subset of the covered lives.
IBM MarketScan Multi-State Medicaid Database (MDCD)	**MDCD_US**	IBM MarketScan Multi-State Medicaid Database (MDCD) contains adjudicated US health insurance claims for Medicaid enrollees from multiple states and includes hospital discharge diagnoses,outpatient diagnoses and procedures, and outpatient pharmacy claims as well as ethnicity andMedicare eligibility. Members maintain their same identifier even if they leave the system for a briefperiod; however the dataset lacks lab data.
IBM MarketScan Commercial Claims and Encounters Database (CCAE)	**CCAE_US**	IBM MarketScan Commercial Claims and Encounters Database (CCAE) is a US employer-based private-payer administrative claims database. The data include adjudicated health insurance claims (e.g. inpatient, outpatient, and outpatient pharmacy) as well as enrollment data from large employers and health plans who provide private healthcare coverage to employees, their spouses, and dependents. Additionally, it captures laboratory tests for a subset of the covered lives. This administrative claims database includes a variety of fee-for-service, preferred provider organizations, and capitated health plans.
Clinical Practice Research Datalink (CPRD)	**CPRD_GOLD_UK**	The Clinical Practice Research Datalink (CPRD) is a governmental, not-for-profit research service, jointly funded by the NHS National Institute for Health Research (NIHR) and the Medicines and Healthcare products Regulatory Agency (MHRA), a part of the Department of Health, United Kingdom (UK). CPRD consists of data collected from UK primary care for all ages. This includes conditions, observations, measurements, and procedures that the general practitioner is made aware of in additional to any prescriptions as prescribed by the general practitioner. In addition to primary care, there are also linked secondary care records for a small number of people. The major data elements contained within this database are outpatient prescriptions given by the general practitioner (coded with Multilex codes) and outpatient clinical, referral, immunization or test events that the general practitioner knows about (coded in Read or ICD10 or LOINC codes). The database also contains the patients' year of births and any date of deaths.

Columbia University Irving Medical Center (CUIMC)	**CUMC_US**	The clinical data warehouse of NewYork-Presbyterian Hospital/Columbia University Irving Medical Center, New York, NY, based on its current and previous electronic health record systems, with data spanning over 30 years and including over 6 million patients
Information System for Research in Primary Care – Hospitalization Linked Data (SIDIAP-H)	**SIDIAP_H_SPAIN**	The Information System for Research in Primary Care (SIDIAP; www.sidiap.org) is a primary care records database from Catalonia, North-East Spain. The SIDIAP-H subset of the database includes around 2 million people out of the total 7 million in SIDIAP that are registered in primary care practices with linked hospital inpatient data available as obtained from the Catalan Institute of Health hospitals. Healthcare is universal and tax-payer funded in the region, and primary care physicians are gatekeepers for all care and responsible for repeat prescriptions.

Appendix Table 2: adverse events of special interest phenotype definition and links

Phenotype name	AESI Target Cohort ID (name and clean period)	Link to ATLAS cohort	Link to package SQL
Acute myocardial infarction IP	312: Acute myocardial infarction IP (365d)	https://atlas.ohdsi.org/#/cohortdefinition/340	https://github.com/ohdsi-studies/Covid19VaccineAesiIncidenceCharacterization/blob/master/inst/sql/sql_server/outcome/AMI_IP.sql
Anaphylaxis	303: Anaphylaxis (30d)	https://atlas.ohdsi.org/#/cohortdefinition/349	https://github.com/ohdsi-studies/Covid19VaccineAesiIncidenceCharacterization/blob/master/inst/sql/sql_server/outcome/Anaphylaxis.sql
Appendicitis	323: Appendicitis (365d)	https://atlas.ohdsi.org/#/cohortdefinition/386	https://github.com/ohdsi-studies/Covid19VaccineAesiIncidenceCharacterization/blob/master/inst/sql/sql_server/outcome/Appendicitis.sql
Bells palsy	307: Bells palsy (183d)	https://atlas.ohdsi.org/#/cohortdefinition/347	https://github.com/ohdsi-studies/Covid19VaccineAesiIncidenceCharacterization/blob/master/inst/sql/sql_server/outcome/BellsPalsy.sql
Deep vein thrombosis	332: Deep vein thrombosis coverICD (365d)	https://atlas.ohdsi.org/#/cohortdefinition/402	https://github.com/ohdsi-studies/Covid19VaccineAesiIncidenceCharacterization/blob/master/inst/sql/sql_server/outcome/DVT_coverICD.sql
Disseminated intravascular coagulation	321: Disseminated intravascular coagulation (365d)	https://atlas.ohdsi.org/#/cohortdefinition/385	https://github.com/ohdsi-studies/Covid19VaccineAesiIncidenceCharacterization/blob/master/inst/sql/sql_server/outcome/DisIntraCoag.sql
Encephalomyelitis IP	308: Encephalomyelitis IP (183d)	https://atlas.ohdsi.org/#/cohortdefinition/346	https://github.com/ohdsi-studies/Covid19VaccineAesiIncidenceCharacterization/blob/master/inst/sql/sql_server/outcome/Encephalomyelitis%20IP.sql
Guillian-Barre syndrome IP	305: Guillian-Barre syndrome IP (365d)	https://atlas.ohdsi.org/#/cohortdefinition/343	https://github.com/ohdsi-studies/Covid19VaccineAesiIncidenceCharacterization/blob/master/inst/sql/sql_server/outcome/GuillainBarreSyndrome%20IP.sql
Hemorrhagic stroke IP	324: Hemorrhagic stroke IP (365d)	https://atlas.ohdsi.org/#/cohortdefinition/405	https://github.com/ohdsi-studies/Covid19VaccineAesiIncidenceCharacterization/blob/master/inst/sql/sql_server/outcome/HemorrhagicStroke%20IP.sql
Immune thrombocytopenia	326: Immune thrombocytopenia (365d)	https://atlas.ohdsi.org/#/cohortdefinition/335	https://github.com/ohdsi-studies/Covid19VaccineAesiIncidenceCharacterization/blob/master/inst/sql/sql_server/outcome/ImmuneThrombocytopenia.sql
Myocarditis pericarditis	314: Myocarditis pericarditis (365d)	https://atlas.ohdsi.org/#/cohortdefinition/339	https://github.com/ohdsi-studies/Covid19VaccineAesiIncidenceCharacterization/blob/master/inst/sql/sql_server/outcome/MyocarditisPericarditis.sql
Narcolepsy	319: Narcolepsy (365d)	https://atlas.ohdsi.org/#/cohortdefinition/345	https://github.com/ohdsi-studies/Covid19VaccineAesiIncidenceCharacterization/blob/master/inst/sql/sql_server/outcome/Narcolepsy.sql
Non-hemorrhagic stroke IP	310: Non-hemorrhagic stroke IP (365d)	https://atlas.ohdsi.org/#/cohortdefinition/406	https://github.com/ohdsi-studies/Covid19VaccineAesiIncidenceCharacterization/blob/master/inst/sql/sql_server/outcome/NonHemorrhagicStroke%20IP.sql

Pulmonary embolism	316: Pulmonary embolism (365d)	https://atlas.ohdsi.org/#/cohortdefinition/411	https://github.com/ohdsi-studies/Covid19VaccineAesiIncidenceCharacterization/blob/master/inst/sql/sql_server/outcome/PulmonaryEmbolism.sql
Transverse myelitis	318: Transverse myelitis (365d)	https://atlas.ohdsi.org/#/cohortdefinition/381	https://github.com/ohdsi-studies/Covid19VaccineAesiIncidenceCharacterization/blob/master/inst/sql/sql_server/outcome/TransverseMyelitis.sql

Appendix Table 3: source clinical codes used in phenotype definition

Phenotype name	ICD10CM	ICD9CM	ICPC-1	KCD7	OXMIS	Read
Acute myocardial infarction IP	I21.9,I21.A,I21.A1,I21.A9,I21,I21.0,I21.1,I21.2,I22,I23,I21.3,I21.4,I22.0,I22.1,I22.8,I22.9,I23.0,I23.1,I23.3,I23.4,I23.6,I23.8,I21.11,I21.29,I21.01,I21.02,I21.21,I21.09,I21.19,I22.2,	410.12,410.2,410.2,410.21,410.7,410.9,410,410.02,410.22,410.32,410.92,410.01,410,410.4,410.71,410.91,410.3,410.8,410.11,410.6,410.72,410.5,410.1,410.62,410,410.3,410.42,410.5,410.82,410.31,410.1,410.4,410.51,410.52,410.6,410.8,410.81,410.41,410.7,410.9,410.61,	K75.00	I21,I21.0,I21.1,I21.2,I21.3,I21.4,I21.9,I22,I22.0,I22.1,I22.8,I22.9,I23.0,I23.1,I23.3,I23.4,I23.6	4109NH, 4109N,4109	G30..00,G304.00,G307000,G30B.00,G380.00,G384.00,G301000,G382.00,G303.00,G307100,G30..13,G30..15,G30..17,G305.00,G364.00,G38z.00,G308.00,G30y200,G30y.00,G351.00,Gyu3600,G353.00,G306.00,G366.00,G30y000,G30..14,G381.00,G30yz00,G30z.00,G35..00,G302.00,G38..00,G30X.00,G383.00,Gyu3500,G31y100,G30..11,G300.00,G301100,G307.00,G301z00,G309.00,G350.00,Gyu3400,G35X.00,G361.00,G301.00,G30X000,G360.00,G363.00,
Anaphylaxis	T78.2,T80.52,T80.59,T80.5,T80.52XA,T78.2XXA,T80.59XD,T78.2XXD,T80.52XD,T80.59XA,	999.4,999.42,995,999.49	A12.01	T78.2,T80.5		SP34.00,SN50.00,SN50.11
Appendicitis	K35.2,K35.3,K35,K35.8,K35.20,K35.21,K35.30,K35.31,K35.32,K35.33,K35.890,K35.891,K36,K37,K35.80,K35.89,	540.9,542,540,540.1,541		K35, K35.2,K35.3, K35.8,K36,K37,	5409B,542 R,5409,542 C,542 G,541	A05y000,J20z100,J201.11,J20..00,J200.00,J222.00,J220.00,J22z.00,J201.12,J202.00,J221.00,J223.00,J20z.00,J21..00,J22..00,J203.00,Jyu2000,J201.00,J204.00
Bells palsy	G51.0	351	N91.00	G51.0		F310.00,2BR9.00
Deep vein thrombosis	I82.50,I80.21,I82.21,I82.22,I82.4,I82.41,I82.42,I82.43,I82.44,I82.49,I82.4Y,I82.4Z,I82.5,I82.59,I82.5Y,I82.62,I82.A,I82.A1,I82.B,I82.B1,I82.C,I82.C1,I82.2,I82.3,I82.40,I82.81,I82.413,I82.439,I82.491,I82.591,I82.819,I82.C11,I82.210,I82.211,I82.402,I82.422,I82.499,I82.622,I82.401,I82.592,I82.623,I82.811,I82.A13,I82.421,I82.629,I82.432,I82.442,I80.219,I82.4Z1,I82.A19,I82.220,I82.431,I82.449,I82.B12,I82.409,I82.493,I82.501,I82.509,I82.5Y1,I82.812,I82.B11,I82.4Y2,I82.4Z2,I82.503,I82.599,I82.A11,I82.403,I82.4Y3,I82.5Y9,I82.B13,I82.411,I82.443,I82.5Y2,I82.412,I82.4Z9,I82.502,I82.593,I82.B19,I82.C12,I82.429,I82.433,I82.441,I82.492,I82.A12,I82.C13,I82.221,I82.4Y1,I82.	453.42,453.86,451.81,453.4,453.2,453.84,453.85,453.3,453.41,453.4,453.83,453.9,453.82,453.89,	K94.01	I82.2,I82.3	453 A,453 L,4510A,453 CT	G802000,G823.00,J420y00,L414.12,G801700,G80y600,SP12200,J420.18,G801600,G801B00,G801.12,G801.13,G801A00,G826.00,G801D00,G80y500,G801800,G82z100,G801C00,G80y700,G801F00,J420200,G801.11,G80y400,G822.00,G825.00,G801H00,G801J00,

	621,I82.419,I82.423,I82.4Y9,I82.4Z3,I82.5Y3,I82.813,I82.C19,					
Dissemited intravascular coagulation	D65,P60	286.6,776.2		D65,P60	2870FL	D306.12,Q452.00,D306.00,L091100
Encephalomyelitis IP	G04,G04.0,G04.3,G04.8,G04.9,G05,B05.0,G07,G36.1,G04.00,G05.4,G04.31,G04.39,G04.01,G04.90,G04.30,G04.81,G05.3,G04.02,G04.32,G04.89,G04.91,	55,323.1,56.01,323,323.63,323.9,323.41,323.01,323.61,323.71,323.4,323.62,323.81,323.5,323.82,323.42,323.51,323.6,323.7,323.8,52,323.2,323.72,323,	N71.03, N71.04	A32.1,B01.1,B05.0,G04,G04.0,G04.8,G04.9,G05,G05.0,G05.1,G05.2,G05.8,G07,G36.1,	323 M,9991A,323 A,323 EN	F034C00,A560100,F03..00,F034F00,F035.00,F03y.11,F034300,F034600,F03X.00,F034000,F03z.00,Fyu0500,F034B00,F034J00,F035000,F0...00,Fyu0.00,F034y00,F034500,Fyu0600,F034x00,F034800,F034900,F034z00,F034E00,F035011,F03y.00,Fyu0A00,F034.00,F034700,F03..11,F034200,F03z.11,F034A00,F034D00,F035100,A520.00,F034H00,F03..12,F0y..00,F034G00,F035z00,F212.00,F034100,F0z..00,Fyu0900,A550.00,F034400,
Guillian-Barre syndrome IP	G61.0	357	N94.01	G61.0	354 GB	F370200,F370000,F370z00,F370.00
Hemorrhagic stroke IP	I60,I60.1,I60.2,I60.3,I60.5,I61,I62,I62.0,I60.4,I60.6,I60.7,I60.8,I60.9,I61.0,I61.1,I61.3,I61.5,I61.6,I61.8,I61.9,I62.9,I60.12,I60.10,I60.30,I60.31,I62.00,I60.52,I60.11,I62.01,I60.21,I60.32,I60.51,I60.50,I60.20,I60.22,I62.02,	430,432.9,432	K90.01	I60,I60.1,I60.2,I60.3,I60.4,I60.6,I60.7,I60.8,I60.9,I61.8,I61.9,I62.0,I62.9,	4309,4319CR	G62z.00,G621.00,Gyu6000,G603.00,G622.00,G602.00,G60X.00,Gyu6100,G604.00,G60z.00,Gyu6E00,G60..00,
Immune thrombocytopenia	D69.3,D75.82,D69.41,D69.42,D69.51	289.84,287.32,287.33,287.31,287.41	B83.02	D69.3	2870B,4464TH	D313.13,D314000,D313y00,G756000,D313011,D313100,D313.12,D313012,D313.14,D314300,G756100,42P2.11,D313.15,D313111,D313000,D313.11,
Myocarditis pericarditis	I30,I40,I01.0,I01.2,I09.0,I09.2,I24.1,I30.0,I30.1,I30.8,I30.9,I31.0,I31.1,I32,I40.0,I40.1,I40.8,I40.9,I41,I51.4,I97.0,D86.85,J11.82,A39.52,B26.82,A39.53,B33.23,B33.22,J10.82,M32.12,A38.1,B58.81,	115.13,391.2,422.99,36.43,393,422,74.21,74.23,398,420.9,422,422.91,422.92,423.1,115.03,422.93,423.2,93.81,93.82,115.93,32.82,420.91,422.9,98.83,420,36.41,422.9,130.3,420.9,420.99,391,420,429,		I40,I01.0,I01.2,I09.0,I09.2,I30,I30.0,I30.1,I30.8,I30.9,I31.0,I31.1,I32,I32.0,I32.8,I40.0,I40.1,I40.8,I40.9,I41,I41.0,I41.1,I41.2,I41.8,I51.4,I97.0,I32.1,	0742M,423 A,0742P,3912,3912N,393 R,420 BB,420,428 B,423 AH,3910AN,3910,423 CM,420 BP,420 NF,9971,	G501.00,G520200,G52y200,Gyu5J00,G5y7.00,G50z300,G520000,G520600,G500100,G500400,A742300,G500200,G5y4000,G52y700,G5y8.00,G51z200,G52y000,G532z00,Gyu5H00,AB41300,G52y500,G5y0.00,G1y0.00,G50z400,G50z500,G520300,G52y.00,G532.00,Gyu5L00,A32y100,A364100,G520700,G52y600,Gyu5400,AD03.00,G500300,G50zz00,G52y100,G52y111,Gyu5F00,G012.00,G102.00,A17y100,G50z511,G10..00,G500500,G532100,G50z111,G52z.00,G531z00,AB40300,G50z.00,N000400,G50z200,G531.00,G500z00,G5yA.00,Gyu5000,AB4z300,G520400,SP2y100,A93y100,A17y200,G101.00,G52..00,G520z00,G52y300,G52yz00,

						G53yz11,G010.00,G500.00,Gyu5200,A364300,A93y000,A98y200,G50z100,G52y400,G558300,G500000,G50z000,G520.00,G520100,Gyu5G00,Gyu5300,A742100,G10z.00,G50..00,G500311,G520500,Gyu5K00,
Narcolepsy	G47.4,G47.42,G47.41,G47.429,G47.421,G47.411,G47.419	347,347,347.1,347.1,347.11,347,347.01		G47.4	3470N	F27..00,F271.00,F27z.00
Non-hemorrhagic stroke IP	P91.82,P91.821,P91.822,P91.823,P91.829,G43.6,G43.60,G43.61,I63,I63.0,I63.01,I63.03,I63.1,I63.11,I63.13,I63.2,I63.21,I63.23,I63.3,I63.31,I63.32,I63.33,I63.34,I63.4,I63.41,I63.42,I63.43,I63.44,I63.5,I63.51,I63.52,I63.53,I63.54,I63.81,I63.89,I63.6,I63.8,I63.9,I63.013,I63.033,I63.113,I63.133,I63.213,I63.233,I63.313,I63.323,I63.333,I63.343,I63.413,I63.423,I63.433,I63.443,I63.513,I63.523,I63.533,I63.543,I63.012,I63.09,I63.522,I63.039,I63.111,I63.22,I63.521,I63.239,I63.329,I63.342,I63.421,G43.601,G43.619,I63.219,I63.29,I63.319,I63.431,I63.432,I63.02,I63.20,I63.40,I63.422,I63.442,I63.50,I63.112,I63.311,I63.321,I63.529,I63.532,I63.019,I63.032,I63.119,I63.232,I63.411,I63.10,I63.312,I63.339,I63.132,I63.211,I63.449,I63.139,I63.212,I63.322,I63.331,I63.39,I63.419,I63.031,I63.12,I63.439,I63.541,I63.341,I63.412,I63.512,I63.531,I63.539,I63.30,I63.549,I63.131,I63.332,I63.429,I63.542,I63.00,I63.011,I63.231,I63.441,I63.49,I63.519,I63.59,G43.609,G43.611,I63.19,I63.349,I63.511	346.6,433.01,433.31,290.42,346.6,290.41,346.62,290.4,433.21,290.43,434.11,433.81,346.63,433.11,434.01,434.91,433.91,779.7,346.61,	K90.03, P70.02	F01.0,F01.1,F01.3,I63,I63.0,I63.1,I63.2,I63.3,I63.4,I63.5,I63.6,I63.8,I63.9,	2930	Eu01100,G63..11,Gyu6G00,G676000,G64z.00,Q48E.00,Eu01000,G640000,G64z400,G63y000,Gyu6400,G64z300,E004.11,Q48E.11,C13A.00,Eu01300,G641000,G64z200,G63y100,G64..12,G6W..00,
Pulmory embolism	I26.93,I26.94,I26,I26.0,I26.9	415.1,415.19,415.11,415.13	K93.00	I26,I26.0,I26.9	450 F,450	G401.12,G401000,G402.00,G401.11,G401.00,F037000,F03..13,F037.00,
Transverse myelitis	G37.3	341.2,341.21,341.22		G37.3	323 B	

Appendix Table 4: age-sex specific crude incidence rates and corresponding 95% confidence intervals for each events of each database

AESI		CCAE_US	CCAE_US	CPRD_GOLD_UK	CPRD_GOLD_UK	CUMC_US	CUMC_US	IPCI_NETHERLANDS	IPCI_NETHERLANDS	IQVIA_AUSTRALIA	IQVIA_AUSTRALIA	IQVIA_FRANCE	IQVIA_FRANCE	IQVIA_GERMANY	IQVIA_GERMANY
	Age	Female	Male	Female	Male	Female	Male	Female	Male	Female	Male	Female	Male	Female	Male
Non-hemorrhagic stroke	1 - 5	3.67 (2.52-5.15)	3.92 (2.76-5.41)	NA	NA	17.38 (5.64-40.56)	29.76 (14.27-54.73)	NA	NA	NA	NA	NA	NA	NA	NA
Non-hemorrhagic stroke	6 - 17	3.67 (3.07-4.35)	3.52 (2.94-4.18)	NA	NA	18.73 (11.1-29.61)	15.52 (8.87-25.21)	NA	NA	NA	NA	NA	NA	NA	NA
Non-hemorrhagic stroke	18 - 34	19.76 (18.56-21.01)	14.45 (13.42-15.54)	NA	NA	30.48 (23.84-38.38)	36.85 (26.88-49.31)	NA	NA	NA	NA	NA	NA	NA	NA
Non-hemorrhagic stroke	35 - 54	85.16 (83.11-87.24)	94.22 (91.93-96.54)	NA	NA	69.66 (61.61-78.46)	145.69 (129.1-163.83)	NA	NA	NA	NA	NA	NA	NA	NA
Non-hemorrhagic stroke	55 - 64	209 (204.81-213.26)	297.1 (291.8-302.47)	NA	NA	154.59 (138.07-172.55)	291.82 (264.05-321.71)	NA	NA	NA	NA	NA	NA	NA	NA
Non-hemorrhagic stroke	65 - 74	317.77 (293.51-343.49)	414.46 (384.96-445.63)	NA	NA	248.79 (226.73-272.41)	410.12 (377.28-445.06)	NA	NA	NA	NA	NA	NA	NA	NA
Non-hemorrhagic stroke	75 - 84	NA	NA	NA	NA	491.97 (452.44-534.02)	523.59 (478.07-572.28)	NA	NA	NA	NA	NA	NA	NA	NA
Non-hemorrhagic stroke	85 - 114	NA	NA	NA	NA	943.04 (862.02-1029.62)	694.61 (612.43-784.75)	NA	NA	NA	NA	NA	NA	NA	NA
Acute myocardial infarction	1 - 5	NA	NA	NA	NA	NA	NA	NA	NA	NA	NA	NA	NA	NA	NA
Acute myocardial infarction	6 - 17	0.28 (0.13-0.52)	0.81 (0.54-1.15)	NA	NA	NA	NA	NA	NA	NA	NA	NA	NA	NA	NA
Acute myocardial infarction	18 - 34	8.48 (7.71-9.32)	17.08 (15.96-18.26)	NA	NA	4.65 (2.32-8.33)	22.93 (15.24-33.14)	NA	NA	NA	NA	NA	NA	NA	NA

Acute myocardial infarction	35 - 54	72.89 (70.99-74.82)	177.11 (173.97-180.29)	NA	NA	32.12 (26.74-38.27)	131.59 (115.84-148.88)	NA	NA	NA	NA	NA	NA	NA	NA
Acute myocardial infarction	55 - 64	217.24 (212.97-221.58)	503.96 (497.04-510.95)	NA	NA	106.91 (93.25-122.01)	303.45 (275.12-333.9)	NA	NA	NA	NA	NA	NA	NA	NA
Acute myocardial infarction	65 - 74	296.25 (272.84-321.12)	676.91 (639.01-716.47)	NA	NA	169.86 (151.73-189.56)	351.51 (321.16-383.95)	NA	NA	NA	NA	NA	NA	NA	NA
Acute myocardial infarction	75 - 84	NA	NA	NA	NA	294.27 (263.91-327.18)	467.24 (424.31-513.34)	NA	NA	NA	NA	NA	NA	NA	NA
Acute myocardial infarction	85 - 114	NA	NA	NA	NA	672.82 (604.73-746.47)	626.95 (549.03-712.83)	NA	NA	NA	NA	NA	NA	NA	NA
Deep vein thrombosis	1 - 5	12.44 (10.25-14.97)	19.09 (16.4-22.09)	NA	NA	107.82 (73.26-153.05)	125.12 (90.17-169.12)	NA	NA	NA	NA	NA	NA	5.24 (2.71-9.15)	7.19 (4.26-11.36)
Deep vein thrombosis	6 - 17	20.03 (18.58-21.55)	18.78 (17.41-20.22)	6.21 (4.47-8.39)	1.56 (0.78-2.79)	52.05 (38.63-68.62)	48.53 (36.02-63.98)	8.24 (4.96-12.86)	3.33 (1.44-6.56)	NA	NA	15.37 (10.58-21.59)	6.42 (3.59-10.58)	35.77 (31.52-40.44)	20.34 (17.22-23.87)
Deep vein thrombosis	18 - 34	159.67 (156.24-163.16)	95.23 (92.56-97.96)	85.6 (79.87-91.63)	33.27 (29.78-37.06)	191.98 (174.71-210.5)	214.84 (189.61-242.49)	100.15 (89.41-111.82)	39.32 (32.8-46.75)	78.11 (53.77-109.7)	42 (21.7-73.37)	148.32 (134.83-162.8)	52.67 (43.77-62.84)	240.42 (232.66-248.37)	162.61 (154.4-171.14)
Deep vein thrombosis	35 - 54	450.39 (445.66-455.16)	384.05 (379.43-388.72)	159.47 (152.81-166.35)	136.52 (130.42-142.83)	319.36 (301.83-337.64)	511.03 (479.47-544.12)	255.89 (241.58-270.83)	173.82 (161.68-186.64)	176.71 (145.24-212.99)	159.88 (125.97-200.12)	313.7 (297.36-330.71)	202.01 (187.75-217.07)	394.08 (386.6-401.67)	384.85 (375.55-394.32)
Deep vein thrombosis	55 - 64	670.01 (662.48-677.61)	836.14 (827.2-845.14)	213.34 (202.13-225.02)	214.98 (203.75-226.67)	456.1 (427.35-486.28)	810.06 (763.25-858.99)	247.45 (228.1-267.99)	309.99 (287.94-333.29)	199.84 (153.22-256.18)	289.79 (229.07-361.67)	390.15 (364.16-417.5)	325.35 (300.51-351.69)	470.49 (460.03-481.13)	523.06 (510.44-535.91)
Deep vein thrombosis	65 - 74	823.32 (783.88-864.23)	1034.03 (986.98-1082.75)	386.82 (370.11-404.09)	343.05 (326.91-359.79)	608.5 (573.67-644.89)	884.29 (835.65-935.02)	420.43 (392.65-449.65)	407.46 (379.41-437.02)	425.37 (346.1-517.36)	403.69 (323.34-497.95)	588.03 (554.23-623.36)	431.36 (400.94-463.48)	621.68 (608.15-635.43)	572.55 (558.11-587.28)

Deep vein thrombosis	75 - 84	NA	NA	585.02 (559.2-611.73)	411.57 (387.76-436.45)	919.14 (864.71-976.1)	1000.32 (936.89-1066.92)	635.45 (590.92-682.44)	497.94 (455.08-543.75)	591.98 (468.68-737.79)	441.46 (329.71-578.92)	834.15 (782.63-888.16)	475.83 (432.5-522.33)	863.77 (847.4-880.39)	640.76 (624.99-656.83)
Deep vein thrombosis	85 - 114	NA	NA	591.28 (554.08-630.32)	384.11 (344.98-426.48)	1522.45 (1418.76-1631.7)	1265.79 (1153.69-1385.84)	745.91 (671.83-825.92)	603.12 (515.61-701.22)	622.82 (446.94-844.92)	573.57 (374.68-840.42)	776.57 (708.43-849.49)	568.02 (492.57-651.77)	1120.05 (1086.17-1154.72)	744.65 (709.32-781.29)
Hemorrhagic stroke	1 - 5	7.33 (5.67-9.33)	6.47 (4.95-8.31)	NA	NA	24.33 (9.78-50.13)	38.69 (20.6-66.16)	NA	NA	NA	NA	NA	NA	NA	NA
Hemorrhagic stroke	6 - 17	5.83 (5.06-6.67)	9.56 (8.59-10.61)	NA	NA	16.65 (9.52-27.04)	22.32 (14.15-33.49)	NA	NA	NA	NA	NA	NA	NA	NA
Hemorrhagic stroke	18 - 34	13.21 (12.24-14.24)	20.58 (19.35-21.87)	NA	NA	22.43 (16.8-29.34)	37.67 (27.58-50.25)	NA	NA	NA	NA	NA	NA	NA	NA
Hemorrhagic stroke	35 - 54	33.12 (31.85-34.43)	42.69 (41.16-44.26)	NA	NA	28.78 (23.7-34.63)	58.45 (48.13-70.33)	NA	NA	NA	NA	NA	NA	NA	NA
Hemorrhagic stroke	55 - 64	73.88 (71.4-76.42)	103.04 (99.93-106.22)	NA	NA	57.81 (47.89-69.18)	97.38 (81.64-115.26)	NA	NA	NA	NA	NA	NA	NA	NA
Hemorrhagic stroke	65 - 74	114.07 (99.74-129.87)	150.99 (133.42-170.23)	NA	NA	88.86 (75.89-103.41)	145.23 (125.98-166.58)	NA	NA	NA	NA	NA	NA	NA	NA
Hemorrhagic stroke	75 - 84	NA	NA	NA	NA	155.58 (133.74-179.97)	191.28 (164.21-221.54)	NA	NA	NA	NA	NA	NA	NA	NA
Hemorrhagic stroke	85 - 114	NA	NA	NA	NA	282.84 (239.39-331.89)	271.2 (220.9-329.53)	NA	NA	NA	NA	NA	NA	NA	NA
Pulmonary embolism	1 - 5	NA	1.27 (0.66-2.22)	NA	NA	NA	NA	NA	NA	NA	NA	NA	NA	NA	NA
Pulmonary embolism	6 - 17	3.53 (2.94-4.2)	2.52 (2.04-3.09)	1.03 (0.42-2.13)	NA	NA	12.61 (6.72-21.57)	2.6 (0.95-5.66)	NA	NA	NA	NA	NA	1.96 (1.07-3.29)	NA
Pulmonary embolism	18 - 34	61.12 (59-63.29)	29.56 (28.08-31.09)	29.03 (25.74-32.63)	11.05 (9.09-13.32)	79.61 (68.63-91.84)	62.25 (49.05-77.92)	44.35 (37.31-52.33)	14.74 (10.87-19.54)	16.56 (6.66-34.12)	NA	15.05 (10.98-20.14)	4.71 (2.35-8.42)	34.5 (31.61-37.59)	22.21 (19.25-25.5)
Pulmonary embolism	35 - 54	152.67 (149.92-155.45)	129.53 (126.85-132.25)	50.29 (46.58-54.21)	47.62 (44.05-51.4)	129.1 (118.05-140.9)	153.01 (135.99-171.58)	107.43 (98.23-117.25)	73.45 (65.63-81.94)	69 (49.93-92.94)	60.94 (40.81-87.52)	23.61 (19.29-28.61)	29.86 (24.54-35.99)	63.9 (60.91-66.99)	78.65 (74.49-83)

Pulmonary embolism	55 - 64	222.49 (218.17-226.88)	306.17 (300.79-311.63)	90.33 (83.09-98.03)	109.48 (101.51-117.91)	159.48 (142.68-177.71)	236.16 (211.26-263.2)	128.07 (114.28-143.08)	161.11 (145.34-178.13)	103.02 (70.46-145.43)	118.69 (81.19-167.56)	32.51 (25.35-41.08)	70.64 (59.35-83.46)	95.41 (90.74-100.26)	144.65 (138.06-151.47)
Pulmonary embolism	65 - 74	282.12 (259.3-306.42)	378.08 (349.93-407.89)	169.89 (158.89-181.45)	192.4 (180.36-205.02)	223.71 (202.83-246.17)	262.28 (236.17-290.5)	228.29 (207.96-250.06)	240.52 (219.11-263.46)	161.23 (114.1-221.3)	189.89 (136.27-257.61)	66.85 (55.82-79.44)	94.07 (80.18-109.67)	163.15 (156.27-170.25)	197.55 (189.11-206.26)
Pulmonary embolism	75 - 84	NA	NA	281.45 (263.66-300.13)	262.39 (243.47-282.39)	333.22 (300.85-368.14)	328.21 (292.4-367.19)	317.05 (285.89-350.68)	288.48 (256.12-323.79)	291.48 (207.27-398.46)	364.5 (263.79-490.98)	129 (109.31-151.21)	123.34 (101.83-148.05)	282.65 (273.34-292.2)	254.65 (244.76-264.84)
Pulmonary embolism	85 - 114	NA	NA	298.4 (272.2-326.46)	299.46 (265.05-337.1)	443.95 (389-504.49)	282.05 (230.69-341.43)	289.09 (243.8-340.35)	338.09 (273.54-413.3)	302.85 (184.99-467.72)	396.48 (234.98-626.6)	150.47 (121.45-184.34)	105.82 (74.88-145.24)	313.13 (295.41-331.64)	293.76 (271.77-317.05)
Appendicitis	1 - 5	32.11 (28.52-36.04)	41.58 (37.57-45.91)	11.04 (6.92-16.72)	10.02 (6.21-15.32)	48.67 (26.61-81.66)	44.64 (24.99-73.63)	38.61 (24.99-57)	39.23 (25.85-57.08)	NA	NA	14.56 (6.66-27.64)	31.58 (19.79-47.81)	77.32 (66.35-89.59)	82.73 (71.84-94.8)
Appendicitis	6 - 17	160.32 (156.19-164.53)	215.15 (210.46-219.92)	35.03 (30.72-39.79)	47.6 (42.65-52.97)	172.93 (147.62-201.32)	228.33 (200.07-259.46)	180.67 (163.72-198.89)	199.78 (182.29-218.5)	89.54 (55.42-136.87)	115.86 (76.36-168.58)	181.93 (164.32-200.91)	187.2 (170.06-205.6)	487.49 (471.38-504)	439 (423.98-454.41)
Appendicitis	18 - 34	201.06 (197.21-204.97)	214.2 (210.19-218.28)	35.03 (31.4-38.96)	39.71 (35.89-43.82)	101.21 (88.79-114.89)	199.97 (175.66-226.7)	194.16 (179.08-210.17)	143.92 (131.18-157.57)	47.33 (28.91-73.1)	42 (21.7-73.37)	83.32 (73.29-94.34)	68.09 (57.92-79.53)	280.43 (272.04-289)	301.27 (290.05-312.81)
Appendicitis	35 - 54	166.53 (163.66-169.44)	151.1 (148.21-154.04)	21.48 (19.09-24.1)	22.25 (19.83-24.88)	66.83 (58.95-75.46)	112.76 (98.23-128.85)	120.93 (111.17-131.33)	97.5 (88.46-107.21)	33.69 (20.85-51.49)	16.8 (7.25-33.11)	32.24 (27.15-38)	33.12 (27.5-39.54)	102.87 (99.07-106.77)	116.49 (111.41-121.75)
Appendicitis	55 - 64	137.01 (133.62-140.46)	118.34 (115-121.74)	19.82 (16.51-23.59)	16.28 (13.3-19.72)	61.7 (51.43-73.41)	66.35 (53.49-81.37)	82.63 (71.63-94.84)	75.21 (64.57-87.11)	25.73 (11.11-50.71)	25.94 (10.43-53.45)	18.58 (13.27-25.3)	15.86 (10.78-22.51)	63.78 (59.97-67.77)	68.05 (63.56-72.78)
Appendicitis	65 - 74	109.59 (95.56-125.11)	96.13 (82.22-111.71)	14.16 (11.11-17.77)	15.76 (12.46-19.67)	43.09 (34.22-53.55)	46.25 (35.69-58.94)	79.29 (67.51-92.52)	75.21 (63.47-88.5)	33.9 (14.64-66.8)	NA	12.95 (8.38-19.12)	17.3 (11.67-24.69)	43.77 (40.25-47.53)	45.14 (41.16-49.4)
Appendicitis	75 - 84	NA	NA	11.16 (7.86-15.38)	20.23 (15.24-26.33)	37.8 (27.46-50.74)	36.5 (25.28-51.01)	77.02 (62.09-94.45)	68.98 (53.67-87.3)	NA	NA	11.87 (6.49-19.91)	8.57 (3.7-16.89)	36.12 (32.85-39.63)	34.9 (31.31-38.8)

Appendicitis	85 - 114	NA	NA	10.63 (6.19-17.03)	14.17 (7.55-24.24)	35.79 (21.55-55.9)	29.5 (14.73-52.78)	40.06 (24.47-61.87)	81.69 (51.79-122.58)	NA	NA	8.08 (2.62-18.85)	NA	41.83 (35.52-48.93)	34.2 (26.99-42.74)
Bells palsy	1 - 5	19.33 (16.57-22.43)	14.1 (11.81-16.72)	11.54 (7.32-17.32)	6.21 (3.3-10.61)	20.85 (7.65-45.39)	23.8 (10.28-46.9)	10.81 (4.35-22.27)	14.52 (6.97-26.71)	NA	NA	NA	NA	19.21 (13.96-25.79)	17.57 (12.77-23.59)
Bells palsy	6 - 17	24.45 (22.86-26.13)	20.41 (18.99-21.92)	18.62 (15.51-22.17)	16.29 (13.45-19.55)	51 (37.73-67.42)	33.96 (23.65-47.23)	21.24 (15.72-28.09)	17.9 (12.95-24.11)	NA	NA	8.85 (5.33-13.82)	10.69 (6.92-15.79)	25.25 (21.69-29.22)	25.9 (22.36-29.84)
Bells palsy	18 - 34	59.49 (57.41-61.63)	45.27 (43.44-47.16)	34.82 (31.2-38.74)	28.84 (25.6-32.38)	60.95 (51.4-71.76)	84.34 (68.84-102.29)	41.49 (34.69-49.23)	29.79 (24.16-36.34)	14.19 (5.21-30.89)	41.99 (21.7-73.35)	18.06 (13.57-23.56)	19.69 (14.41-26.26)	28.28 (25.67-31.1)	45.97 (41.65-50.6)
Bells palsy	35 - 54	104.98 (102.71-107.29)	101.38 (99.01-103.79)	37.73 (34.53-41.14)	37.75 (34.58-41.14)	65.01 (57.25-73.54)	82.44 (70.09-96.35)	45.12 (39.24-51.63)	42.77 (36.86-49.35)	22.45 (12.28-37.67)	46.22 (28.96-69.97)	30.64 (25.69-36.27)	35.01 (29.23-41.6)	35.3 (33.09-37.62)	51.88 (48.51-55.43)
Bells palsy	55 - 64	124.06 (120.84-127.35)	134.71 (131.15-138.34)	36.8 (32.24-41.83)	46.17 (41.05-51.75)	77.23 (65.69-90.21)	98.07 (82.28-116.01)	62.14 (52.66-72.84)	60.82 (51.29-71.61)	38.6 (19.94-67.42)	51.89 (28.37-87.06)	33.9 (26.58-42.63)	40.42 (32-50.37)	44.35 (41.19-47.7)	59.88 (55.67-64.33)
Bells palsy	65 - 74	128.04 (112.83-144.72)	151.49 (133.89-170.75)	33.09 (28.35-38.41)	44.24 (38.58-50.51)	89.37 (76.37-103.96)	106.02 (89.68-124.47)	74.33 (62.95-87.18)	53.92 (44.06-65.33)	42.37 (20.32-77.92)	36.98 (15.97-72.87)	32.63 (25.08-41.75)	32.87 (24.89-42.58)	50.55 (46.75-54.57)	66.37 (61.52-71.49)
Bells palsy	75 - 84	NA	NA	43.74 (36.91-51.47)	40.45 (33.25-48.76)	125.44 (105.92-147.52)	119.21 (98.06-143.55)	91.21 (74.9-110.03)	76.95 (60.73-96.18)	67.09 (30.68-127.36)	50.68 (18.6-110.31)	26.28 (17.86-37.3)	20.35 (12.25-31.79)	67.3 (62.8-72.03)	67.57 (62.52-72.91)
Bells palsy	85 - 114	NA	NA	23.77 (16.82-32.63)	42.53 (30.24-58.14)	129.99 (101.14-164.51)	93.88 (65.39-130.57)	58.08 (38.9-83.41)	92.33 (60.31-135.28)	NA	NA	25.85 (14.78-41.99)	30.6 (15.28-54.75)	93.84 (84.27-104.21)	91.94 (79.84-105.35)
Anaphylaxis	1 - 5	80.77 (75-86.86)	116.76 (109.96-123.87)	12.55 (8.12-18.52)	16.71 (11.64-23.23)	125.11 (87.63-173.21)	241.03 (191.41-299.58)	NA	39.22 (25.84-57.06)	427.02 (293.94-599.69)	397.57 (275.33-555.56)	NA	NA	41.48 (33.56-50.7)	45.93 (37.92-55.13)
Anaphylaxis	6 - 17	82.87 (79.91-85.91)	99.84 (96.65-103.1)	11.67 (9.24-14.55)	12.32 (9.87-15.2)	131.13 (109.23-156.13)	141.66 (119.61-166.59)	23.84 (17.96-31.04)	25.81 (19.79-33.08)	682.04 (580.45-796.29)	767.98 (659.59-889.09)	NA	2.14 (0.69-4.99)	27.21 (23.51-31.32)	46.24 (41.46-51.41)
Anaphylaxis	18 - 34	81.36 (78.92-83.86)	45.86 (44.01-47.76)	17.25 (14.73-20.07)	13.57 (11.37-16.06)	52.05 (43.26-62.1)	47.49 (36.06-61.39)	25.96 (20.65-32.23)	17.81 (13.52-23.02)	151.4 (116.6-193.34)	150.47 (108.89-202.68)	NA	2.57 (0.94-5.59)	20.68 (18.45-23.11)	23.53 (20.48-26.91)
Anaphylaxis	35 - 54	76.86 (74.92-78.85)	41.9 (40.39-43.46)	16.39 (14.3-18.69)	8.26 (6.81-9.92)	32.63 (27.2-38.82)	24.52 (18.01-32.6)	20.52 (16.62-25.06)	17.38 (13.69-21.75)	129.91 (103.16-161.46)	71.41 (49.46-99.79)	3.86 (2.25-6.18)	1.9 (0.76-3.91)	23.95 (22.14-25.88)	21.78 (19.61-24.11)

Anaphylaxis	55 - 64	60.83 (58.58-63.14)	43.73 (41.71-45.82)	16.83 (13.79-20.33)	10.17 (7.85-12.96)	23.79 (17.6-31.46)	10.81 (6.05-17.83)	41.69 (33.99-50.61)	29.56 (23.04-37.34)	138.3 (100.09-186.29)	62.99 (36.7-100.86)	3.71 (1.6-7.32)	NA	23.91 (21.6-26.4)	19.72 (17.33-22.34)
Anaphylaxis	65 - 74	54.99 (45.19-66.27)	40.1 (31.32-50.59)	12.62 (9.76-16.06)	9.9 (7.32-13.08)	14.36 (9.46-20.89)	12.09 (7.04-19.36)	26.08 (19.54-34.11)	52.35 (42.64-63.61)	131.34 (89.24-186.43)	87.82 (52.87-137.14)	8.29 (4.74-13.46)	NA	19.64 (17.31-22.21)	15.77 (13.46-18.37)
Anaphylaxis	75 - 84	NA	NA	8.45 (5.61-12.21)	9.19 (5.95-13.57)	21.47 (13.89-31.69)	10.73 (5.15-19.74)	25.09 (16.93-35.82)	27.97 (18.59-40.43)	134.14 (79.5-212)	59.12 (23.77-121.8)	NA	NA	15.06 (12.97-17.39)	10.38 (8.46-12.6)
Anaphylaxis	85 - 114	NA	NA	6.25 (3-11.5)	NA	11.3 (4.15-24.59)	NA	12.01 (4.41-26.15)	NA	NA	NA	NA	NA	10.99 (7.89-14.91)	6.22 (3.4-10.43)
Immune thrombocytopenia	1 - 5	13.78 (11.46-16.43)	19.94 (17.19-23)	8.03 (4.59-13.04)	13.37 (8.88-19.32)	38.24 (19.09-68.42)	29.76 (14.27-54.73)	10.81 (4.35-22.27)	13.07 (5.98-24.82)	NA	NA	NA	NA	8.73 (5.33-13.49)	15.98 (11.42-21.76)
Immune thrombocytopenia	6 - 17	11.62 (10.53-12.8)	10.15 (9.16-11.23)	4.43 (2.99-6.33)	5.1 (3.57-7.06)	41.64 (29.75-56.7)	35.91 (25.28-49.49)	6.94 (3.97-11.27)	6.66 (3.81-10.82)	NA	NA	NA	NA	5.61 (4.01-7.64)	5.15 (3.65-7.07)
Immune thrombocytopenia	18 - 34	31.85 (30.33-33.43)	13.18 (12.2-14.22)	4.55 (3.3-6.1)	2.31 (1.47-3.47)	52.07 (43.28-62.13)	39.31 (28.98-52.12)	8.55 (5.63-12.44)	1.84 (0.68-4.01)	NA	NA	3.34 (1.6-6.15)	NA	3.63 (2.74-4.73)	3.98 (2.79-5.51)
Immune thrombocytopenia	35 - 54	39.87 (38.48-41.31)	26.23 (25.04-27.47)	3.84 (2.87-5.03)	2.17 (1.47-3.1)	42.4 (36.18-49.39)	38.61 (30.32-48.48)	10.26 (7.57-13.61)	5.49 (3.52-8.17)	9.62 (3.53-20.94)	NA	1.36 (0.5-2.96)	NA	4.02 (3.3-4.85)	4.07 (3.17-5.15)
Immune thrombocytopenia	55 - 64	47.7 (45.71-49.76)	52.08 (49.87-54.35)	5.98 (4.23-8.2)	5.63 (3.95-7.8)	32.06 (24.79-40.78)	53.36 (41.9-66.99)	11.85 (7.94-17.02)	13.09 (8.89-18.58)	NA	NA	NA	NA	4.39 (3.44-5.53)	4.39 (3.31-5.71)
Immune thrombocytopenia	65 - 74	44.51 (35.75-54.77)	65 (53.66-78.02)	8.22 (5.95-11.08)	8.69 (6.29-11.7)	38.3 (29.97-48.23)	74.73 (61.12-90.46)	12.8 (8.36-18.75)	18.14 (12.64-25.23)	NA	NA	3.11 (1.14-6.76)	NA	6.39 (5.09-7.93)	6.6 (5.13-8.35)
Immune thrombocytopenia	75 - 84	NA	NA	8.14 (5.37-11.85)	17.28 (12.7-22.98)	56.7 (43.85-72.14)	78.41 (61.46-98.58)	13.39 (7.65-21.74)	24.98 (16.17-36.88)	NA	NA	6.78 (2.93-13.36)	NA	5.18 (3.99-6.62)	10.07 (8.19-12.26)
Immune thrombocytopenia	85 - 114	NA	NA	8.13 (4.33-13.9)	18.54 (10.8-29.68)	65.94 (45.93-91.7)	75.12 (49.91-108.56)	18.02 (8.24-34.22)	39.05 (19.5-69.88)	NA	NA	NA	NA	6.7 (4.34-9.89)	9.77 (6.12-14.79)
Myocarditis pericarditis	1 - 5	5.33 (3.93-7.07)	6.26 (4.76-8.07)	NA	NA	52.15 (29.19-86.02)	62.5 (38.69-95.54)	NA	NA	NA	NA	NA	NA	4.8 (2.4-8.59)	7.19 (4.26-11.36)
Myocarditis pericarditis	6 - 17	6.64 (5.82-7.54)	12.25 (11.15-13.43)	NA	3.82 (2.52-5.56)	42.68 (30.63-57.9)	44.64 (32.68-59.55)	NA	NA	NA	NA	NA	3.42 (1.48-6.74)	13.75 (11.16-16.75)	16 (13.24-19.16)
Myocarditis pericarditis	18 - 34	21.68 (20.42-22.98)	39.9 (38.18-41.68)	7.54 (5.91-9.48)	23.32 (20.41-26.52)	34.29 (27.23-42.61)	93.4 (77.05-112.21)	NA	NA	NA	NA	8.03 (5.14-11.94)	17.55 (12.59-23.81)	25.18 (22.72-27.84)	61.9 (56.88-67.25)

Myocarditis pericarditis	35 - 54	36.83 (35.49-38.21)	46.42 (44.82-48.06)	9.52 (7.95-11.32)	24.42 (21.88-27.17)	37.78 (31.92-44.4)	83 (70.6-96.95)	NA	NA	NA	NA	9.99 (7.26-13.41)	16.83 (12.9-21.57)	28.61 (26.62-30.71)	53.61 (50.18-57.21)
Myocarditis pericarditis	55 - 64	50.26 (48.22-52.37)	69.09 (66.55-71.7)	11.17 (8.72-14.08)	25.67 (21.89-29.91)	49.55 (40.4-60.15)	103.89 (87.61-122.31)	NA	NA	NA	NA	16.26 (11.32-22.61)	10.74 (6.65-16.42)	30.02 (27.42-32.79)	44.64 (41.02-48.5)
Myocarditis pericarditis	65 - 74	67.52 (56.61-79.92)	75.75 (63.46-89.71)	12.82 (9.93-16.28)	20.81 (16.98-25.24)	51.61 (41.85-62.96)	101.08 (85.14-119.14)	NA	NA	NA	NA	5.18 (2.48-9.53)	8.07 (4.41-13.54)	22.34 (19.85-25.07)	26.11 (23.1-29.39)
Myocarditis pericarditis	75 - 84	NA	NA	9.95 (6.85-13.98)	21.7 (16.52-27.99)	48.11 (36.34-62.47)	76.26 (59.56-96.19)	NA	NA	NA	NA	5.09 (1.87-11.07)	9.64 (4.41-18.3)	20.73 (18.27-23.43)	23.4 (20.47-26.63)
Myocarditis pericarditis	85 - 114	NA	NA	6.88 (3.43-12.31)	6.54 (2.4-14.24)	32.03 (18.66-51.28)	40.23 (22.52-66.35)	NA	NA	NA	NA	NA	NA	12.87 (9.49-17.06)	12.88 (8.62-18.49)
Disseminated intravascular coagulation	1 - 5	1.78 (1.02-2.89)	2.76 (1.8-4.04)	NA	NA	NA	20.83 (8.38-42.92)	NA	NA	NA	NA	NA	NA	NA	NA
Disseminated intravascular coagulation	6 - 17	1.65 (1.26-2.13)	2.26 (1.8-2.79)	NA	NA	11.45 (5.71-20.48)	9.7 (4.65-17.84)	NA	NA	NA	NA	NA	NA	NA	NA
Disseminated intravascular coagulation	18 - 34	5.02 (4.42-5.67)	3.04 (2.58-3.56)	NA	NA	10.58 (6.85-15.62)	10.64 (5.67-18.2)	NA	NA	NA	NA	NA	NA	0.53 (0.23-1.04)	NA
Disseminated intravascular coagulation	35 - 54	7.3 (6.71-7.93)	6.14 (5.57-6.76)	NA	NA	7.96 (5.41-11.3)	15.13 (10.13-21.73)	NA	NA	NA	NA	NA	NA	NA	NA
Disseminated intravascular coagulation	55 - 64	13.12 (12.09-14.22)	14.44 (13.3-15.67)	NA	NA	12.14 (7.86-17.92)	25.95 (18.18-35.93)	NA	NA	NA	NA	NA	NA	0.43 (0.17-0.88)	0.72 (0.33-1.36)
Disseminated intravascular coagulation	65 - 74	16 (10.94-22.58)	24.86 (18.06-33.37)	NA	NA	21.81 (15.65-29.58)	30.59 (22.14-41.2)	NA	NA	NA	NA	NA	NA	0.69 (0.32-1.32)	NA
Disseminated intravascular coagulation	75 - 84	NA	NA	NA	NA	24.91 (16.68-35.77)	28.98 (19.1-42.17)	NA	NA	NA	NA	NA	NA	0.73 (0.33-1.38)	1.73 (1.01-2.77)
Disseminated intravascular coagulation	85 - 114	NA	NA	NA	NA	35.78 (21.54-55.88)	37.54 (20.52-62.99)	NA	NA	NA	NA	NA	NA	NA	NA
Encephalomyelitis	1 - 5	4.55 (3.27-6.18)	5.51 (4.12-7.23)	NA	NA	NA	14.88 (4.83-34.72)	NA	NA	NA	NA	NA	NA	NA	NA
Encephalomyelitis	6 - 17	4.59 (3.92-5.35)	4.73 (4.05-5.48)	NA	NA	20.81 (12.71-32.15)	22.32 (14.15-33.48)	NA	NA	NA	NA	NA	NA	NA	NA

Encephalomyelitis	18 - 34	4.59 (4.02-5.21)	4.61 (4.03-5.24)	NA	NA	10.58 (6.85-15.62)	13.1 (7.49-21.27)	NA	NA	NA	NA	NA	NA	NA	NA
Encephalomyelitis	35 - 54	5.56 (5.05-6.12)	5.11 (4.59-5.67)	NA	NA	6.42 (4.16-9.48)	12.52 (8.02-18.63)	NA	NA	NA	NA	NA	NA	NA	NA
Encephalomyelitis	55 - 64	9.68 (8.8-10.63)	10.75 (9.76-11.81)	NA	NA	8.26 (4.81-13.22)	18.02 (11.66-26.6)	NA	NA	NA	NA	NA	NA	NA	NA
Encephalomyelitis	65 - 74	14 (9.3-20.23)	16.38 (10.97-23.53)	NA	NA	10.64 (6.5-16.43)	16.36 (10.37-24.55)	NA	NA	NA	NA	NA	NA	NA	NA
Encephalomyelitis	75 - 84	NA	NA	NA	NA	9.45 (4.72-16.9)	15.03 (8.22-25.21)	NA	NA	NA	NA	NA	NA	NA	NA
Encephalomyelitis	85 - 114	NA	NA	NA	NA	9.42 (3.06-21.97)	13.41 (4.35-31.29)	NA	NA	NA	NA	NA	NA	NA	NA
Narcolepsy	1 - 5	1.22 (0.61-2.19)	0.53 (0.17-1.24)	NA	NA	NA	NA	NA	NA	NA	NA	NA	NA	NA	NA
Narcolepsy	6 - 17	12.35 (11.22-13.56)	10.1 (9.1-11.17)	0.89 (0.33-1.93)	0.99 (0.4-2.04)	13.53 (7.2-23.14)	17.46 (10.35-27.6)	NA	NA	NA	NA	NA	NA	4.07 (2.72-5.84)	3.8 (2.52-5.49)
Narcolepsy	18 - 34	61.43 (59.31-63.61)	36.74 (35.08-38.45)	2.58 (1.67-3.81)	0.7 (0.28-1.45)	33.44 (26.47-41.68)	41.77 (31.1-54.91)	NA	NA	11.83 (3.84-27.6)	NA	4.01 (2.07-7.01)	3.85 (1.76-7.31)	6.28 (5.08-7.67)	10.72 (8.69-13.07)
Narcolepsy	35 - 54	58.15 (56.46-59.88)	37.4 (35.97-38.88)	1.62 (1.02-2.46)	1.16 (0.66-1.88)	21.07 (16.76-26.15)	20.35 (14.47-27.82)	NA	NA	9.62 (3.53-20.94)	NA	1.59 (0.64-3.27)	1.9 (0.76-3.91)	4.91 (4.11-5.82)	8.44 (7.11-9.94)
Narcolepsy	55 - 64	40.51 (38.68-42.4)	33.48 (31.72-35.32)	1.42 (0.65-2.69)	NA	9.23 (5.56-14.41)	11.53 (6.59-18.73)	NA	NA	NA	18.53 (6.02-43.24)	NA	NA	4.27 (3.33-5.39)	7.42 (5.99-9.1)
Narcolepsy	65 - 74	33.5 (25.96-42.55)	35.03 (26.86-44.91)	NA	NA	7.45 (4.07-12.49)	14.94 (9.25-22.83)	NA	NA	NA	NA	NA	NA	3.16 (2.27-4.28)	4.21 (3.06-5.65)
Narcolepsy	75 - 84	NA	NA	NA	NA	11.16 (5.94-19.09)	8.59 (3.71-16.92)	NA	NA	NA	NA	NA	NA	2.91 (2.04-4.04)	5.49 (4.13-7.17)
Narcolepsy	85 - 114	NA	NA	NA	NA	13.18 (5.3-27.16)	NA	NA	NA	NA	NA	NA	NA	3.75 (2.05-6.3)	3.11 (1.25-6.4)
Guillain-Barre syndrome	1 - 5	1.11 (0.53-2.04)	1.48 (0.81-2.49)	NA	NA	NA	NA	NA	NA	NA	NA	NA	NA	NA	NA

Guillain-Barre syndrome	6 - 17	0.78 (0.52-1.13)	0.64 (0.41-0.96)	NA	NA	NA	NA	NA	NA	NA	NA	NA	NA	NA	NA
Guillain-Barre syndrome	18 - 34	2.87 (2.42-3.37)	2.11 (1.73-2.56)	NA	NA	NA	6.55 (2.83-12.91)	NA	NA	NA	NA	NA	NA	NA	NA
Guillain-Barre syndrome	35 - 54	3.57 (3.16-4.01)	3.67 (3.23-4.15)	NA	NA	NA	3.13 (1.15-6.81)	NA	NA	NA	NA	NA	NA	NA	NA
Guillain-Barre syndrome	55 - 64	4.57 (3.96-5.23)	6.86 (6.08-7.72)	NA	NA	3.88 (1.68-7.65)	10.81 (6.05-17.84)	NA	NA	NA	NA	NA	NA	NA	NA
Guillain-Barre syndrome	65 - 74	6 (3.1-10.48)	9.04 (5.17-14.68)	NA	NA	4.79 (2.19-9.09)	4.27 (1.57-9.29)	NA	NA	NA	NA	NA	NA	NA	NA
Guillain-Barre syndrome	75 - 84	NA	NA	NA	NA	4.29 (1.39-10.02)	NA	NA	NA	NA	NA	NA	NA	NA	NA
Guillain-Barre syndrome	85 - 114	NA	NA	NA	NA	NA	NA	NA	NA	NA	NA	NA	NA	NA	NA
Transverse myelitis	1 - 5	1.11 (0.53-2.04)	0.95 (0.44-1.81)	NA	NA	NA	NA	NA	NA	NA	NA	NA	NA	NA	NA
Transverse myelitis	6 - 17	1.34 (0.99-1.78)	1.34 (1-1.77)	NA	NA	5.2 (1.69-12.14)	4.85 (1.58-11.32)	NA	NA	NA	NA	NA	NA	NA	NA
Transverse myelitis	18 - 34	5.58 (4.95-6.26)	2.61 (2.18-3.09)	0.93 (0.43-1.76)	NA	8.89 (5.5-13.58)	5.73 (2.3-11.81)	NA	NA	NA	NA	NA	NA	NA	NA
Transverse myelitis	35 - 54	10.65 (9.93-11.4)	5.73 (5.18-6.33)	1.85 (1.19-2.72)	1.23 (0.72-1.97)	8.48 (5.84-11.91)	10.96 (6.78-16.75)	NA	NA	NA	NA	NA	NA	0.6 (0.34-0.97)	0.53 (0.24-1.01)
Transverse myelitis	55 - 64	9.51 (8.63-10.45)	7.73 (6.9-8.64)	1.73 (0.86-3.09)	NA	5.34 (2.67-9.56)	5.77 (2.49-11.36)	NA	NA	NA	NA	NA	NA	0.55 (0.25-1.04)	0.4 (0.13-0.93)
Transverse myelitis	65 - 74	7 (3.83-11.74)	7.34 (3.91-12.56)	0.96 (0.31-2.23)	1.41 (0.57-2.91)	4.25 (1.84-8.38)	4.27 (1.57-9.29)	NA	NA	NA	NA	NA	NA	NA	0.57 (0.21-1.25)
Transverse myelitis	75 - 84	NA	NA	NA	NA	NA	5.37 (1.74-12.53)	NA	NA	NA	NA	NA	NA	NA	NA
Transverse myelitis	85 - 114	NA	NA	NA	NA	NA	NA	NA	NA	NA	NA	NA	NA	NA	NA

Cont

AESI		JMDC_JAPAN	JMDC_JAPAN	MDCD_US	MDCD_US	MDCR_US	MDCR_US	OPTUM_EHR_US	OPTUM_EHR_US	OPTUM_SES_US	OPTUM_SES_US	SIDIAP_H_SPAIN	SIDIAP_H_SPAIN
	Age	Female	Male	Female	Male	Female	Male	Female	Male	Female	Male	Female	Male
Non-hemorrhagic stroke	1 - 5	2.27 (0.98-4.47)	4.29 (2.45-6.97)	4.19 (3.17-5.44)	5.82 (4.63-7.23)	NA	NA	4.69 (3.57-6.06)	6.38 (5.11-7.86)	4.19 (2.48-6.63)	3.31 (1.85-5.46)	NA	NA
Non-hemorrhagic stroke	6 - 17	2.41 (1.57-3.52)	3.95 (2.88-5.28)	5.23 (4.54-6)	5.04 (4.39-5.77)	NA	NA	4.08 (3.48-4.75)	4.66 (4.03-5.37)	2.97 (2.19-3.96)	4 (3.1-5.09)	NA	2.33 (1.01-4.59)
Non-hemorrhagic stroke	18 - 34	7.53 (5.99-9.34)	8.04 (6.73-9.53)	45.04 (42.69-47.49)	42.76 (39.65-46.04)	NA	NA	14.39 (13.6-15.21)	17.05 (15.97-18.19)	22.24 (20.4-24.2)	18.84 (17.21-20.58)	8.44 (6.03-11.5)	7.94 (5.59-10.94)
Non-hemorrhagic stroke	35 - 54	40.61 (37.99-43.37)	66.24 (63.25-69.33)	305.32 (298.36-312.41)	387.53 (376.97-398.31)	NA	NA	65.71 (64.26-67.19)	99.39 (97.22-101.61)	134.03 (130.29-137.85)	130.65 (127.04-134.34)	40.62 (36.5-45.07)	72.27 (66.8-78.07)
Non-hemorrhagic stroke	55 - 64	111.57 (103.88-119.67)	242.98 (233.46-252.8)	846.22 (829.14-863.57)	1100.23 (1077.7-1123.11)	NA	NA	150.29 (147.41-153.21)	251.86 (247.57-256.21)	442.01 (433.01-451.16)	539.42 (529.39-549.59)	112.99 (102.1-124.71)	303.28 (285.31-322.08)
Non-hemorrhagic stroke	65 - 74	307.55 (283.37-333.24)	564.28 (535.34-594.38)	1027.72 (1004.85-1050.98)	1342.11 (1307.49-1377.41)	597.68 (578.86-616.96)	794.61 (771.32-818.43)	264.29 (259.72-268.92)	377.95 (371.76-384.23)	818.31 (808.54-828.17)	994.07 (981.96-1006.29)	258.46 (240.81-277.07)	511.83 (486.11-538.56)
Non-hemorrhagic stroke	75 - 84	794.37 (553.31-1104.78)	1153.98 (888.71-1473.61)	1401.44 (1366.13-1437.43)	1606.03 (1547.38-1666.32)	1345.43 (1312.72-1378.76)	1594.88 (1555.47-1635.03)	555.68 (546.69-564.79)	655.47 (644.31-666.77)	1525.28 (1509.31-1541.39)	1739.54 (1720.05-1759.2)	614.73 (583.01-647.74)	868.74 (827.19-911.85)
Non-hemorrhagic stroke	85 - 114	NA	NA	1882.06 (1819.71-1946)	1793.43 (1673.77-1919.4)	2699.42 (2635.26-2764.75)	2862.26 (2777.61-2948.84)	1068.56 (1050.76-1086.59)	1010.5 (988.15-1033.23)	2652.97 (2622.79-2683.4)	2766.21 (2725.77-2807.1)	914.96 (865.84-966.14)	1109.01 (1036.61-1185.12)
Acute myocardial infarction	1 - 5	NA	NA	NA	0.43 (0.16-0.93)	NA	NA	NA	0.58 (0.25-1.14)	NA	NA	NA	NA
Acute myocardial infarction	6 - 17	NA	0.61 (0.25-1.27)	0.44 (0.25-0.7)	0.74 (0.5-1.05)	NA	NA	0.52 (0.32-0.79)	0.72 (0.48-1.02)	0.82 (0.44-1.41)	1.09 (0.65-1.72)	NA	NA
Acute myocardial infarction	18 - 34	1.19 (0.64-2.04)	2.24 (1.57-3.08)	31.59 (29.62-33.65)	51.17 (47.76-54.75)	NA	NA	10.75 (10.07-11.46)	28.01 (26.62-29.46)	9.01 (7.85-10.29)	20.68 (18.97-22.5)	2.11 (1.01-3.88)	9.01 (6.5-12.18)
Acute myocardial infarction	35 - 54	7.01 (5.95-8.21)	45.18 (42.72-47.74)	296.5 (289.64-303.48)	542.91 (530.38-555.65)	NA	NA	75.63 (74.07-77.21)	197.37 (194.29-200.48)	101.32 (98.07-104.66)	205.92 (201.37-210.54)	33.64 (29.9-37.71)	187.88 (178.99-197.1)
Acute myocardial infarction	55 - 64	23.66 (20.2-27.55)	135.73 (128.64-143.11)	849 (831.89-866.38)	1203.14 (1179.56-1227.07)	NA	NA	189.78 (186.55-193.06)	449.96 (444.21-455.76)	389.98 (381.52-398.58)	735.69 (723.96-747.57)	118.74 (107.58-130.75)	577.57 (552.63-603.33)

Acute myocardial infarction	65 - 74	65.28 (54.42-77.67)	216.26 (198.51-235.16)	970.27 (948.07-992.87)	1326.08 (1291.68-1361.16)	564.11 (545.83-582.85)	1043.02 (1016.28-1070.28)	320.81 (315.78-325.91)	601.49 (593.66-609.4)	715.71 (706.58-724.93)	1172.53 (1159.36-1185.81)	204.76 (189.08-221.39)	646.44 (617.47-676.42)
Acute myocardial infarction	75 - 84	NA	268.38 (150.21-442.66)	1251.55 (1218.22-1285.56)	1585.65 (1527.42-1645.52)	1136.06 (1106.05-1166.69)	1760.2 (1718.78-1802.37)	606.73 (597.33-616.23)	904.75 (891.62-918.02)	1234.26 (1219.91-1248.74)	1807.26 (1787.39-1827.3)	357.78 (333.69-383.15)	767.92 (728.88-808.5)
Acute myocardial infarction	85 - 114	NA	NA	1555.34 (1498.79-1613.47)	2024.13 (1896.86-2157.71)	2232.63 (2174.44-2291.98)	3121.63 (3033.2-3211.98)	1086.47 (1068.52-1104.65)	1400.11 (1373.74-1426.85)	2078.63 (2052.01-2105.52)	2885.13 (2843.84-2926.86)	418.5 (385.54-453.51)	737.42 (678.67-799.9)
Deep vein thrombosis	1 - 5	15.88 (11.99-20.62)	20.11 (15.82-25.21)	16.99 (14.85-19.35)	21.02 (18.69-23.56)	NA	NA	9.15 (7.55-10.98)	12.54 (10.74-14.55)	17 (13.33-21.38)	20.51 (16.56-25.13)	6.46 (2.37-14.06)	NA
Deep vein thrombosis	6 - 17	28.41 (25.32-31.77)	30.27 (27.16-33.64)	20.81 (19.4-22.29)	19.08 (17.78-20.45)	NA	NA	14.67 (13.52-15.89)	14.4 (13.27-15.6)	19.56 (17.44-21.86)	19.89 (17.8-22.16)	7.38 (4.68-11.07)	7.57 (4.94-11.09)
Deep vein thrombosis	18 - 34	151.3 (144.08-158.79)	45.96 (42.75-49.35)	278.72 (272.82-284.72)	210.42 (203.46-217.57)	NA	NA	149.48 (146.92-152.07)	131.53 (128.49-134.62)	161.02 (156-166.16)	97.61 (93.85-101.48)	65.26 (58.18-72.95)	42.29 (36.59-48.62)
Deep vein thrombosis	35 - 54	212.77 (206.7-218.96)	117.15 (113.16-121.24)	866.16 (854.37-878.07)	957.1 (940.42-974.01)	NA	NA	352.27 (348.9-355.66)	419.84 (415.35-424.37)	490.53 (483.33-497.81)	401.58 (395.21-408.01)	158.88 (150.62-167.46)	143.63 (135.87-151.71)
Deep vein thrombosis	55 - 64	335.09 (321.66-348.93)	275.26 (265.11-285.69)	1352.08 (1330.4-1374.02)	1480.05 (1453.84-1506.6)	NA	NA	491.33 (486.11-496.59)	734.1 (726.75-741.52)	930.75 (917.63-944.02)	1056.15 (1042.06-1070.38)	288.41 (270.84-306.82)	325.69 (307.06-345.16)
Deep vein thrombosis	65 - 74	579.46 (546.04-614.38)	477.89 (451.3-505.64)	1442.76 (1415.58-1470.33)	1509.52 (1472.75-1546.98)	1371.23 (1342.54-1400.38)	1557.05 (1524.26-1590.37)	711.06 (703.54-718.64)	901.5 (891.89-911.18)	1325.56 (1313.08-1338.12)	1494.65 (1479.75-1509.66)	502.34 (477.57-528.06)	441.3 (417.44-466.16)
Deep vein thrombosis	75 - 84	726.94 (497.23-1026.23)	664.03 (467.54-915.28)	1747.85 (1708.3-1788.08)	1690.03 (1629.8-1751.91)	2167.67 (2125.91-2210.04)	2256.26 (2209.19-2304.08)	1071.35 (1058.82-1083.99)	1172.54 (1157.56-1187.66)	1953.58 (1935.45-1971.84)	2030.21 (2009.11-2051.48)	807.95 (771.46-845.72)	601.31 (566.84-637.32)
Deep vein thrombosis	85 - 114	NA	NA	2050.77 (1985.51-2117.62)	1870.07 (1747.64-1998.8)	2920.22 (2853.28-2988.33)	3033.06 (2945.72-3122.34)	1422.23 (1401.64-1443.04)	1386.23 (1359.98-1412.86)	2648.7 (2618.51-2679.16)	2596.45 (2557.24-2636.12)	879.49 (831.29-929.75)	647.58 (592.59-706.3)
Hemorrhagic stroke	1 - 5	1.98 (0.8-4.09)	2.95 (1.47-5.28)	12.43 (10.61-14.47)	14.98 (13.03-17.15)	NA	NA	5.57 (4.34-7.04)	6.67 (5.37-8.18)	6.75 (4.52-9.7)	7.94 (5.56-10.99)	NA	NA
Hemorrhagic stroke	6 - 17	2.13 (1.35-3.19)	4.74 (3.56-6.18)	7.9 (7.04-8.84)	12.17 (11.13-13.27)	NA	NA	3.71 (3.14-4.35)	5.64 (4.95-6.41)	5.57 (4.47-6.86)	10.98 (9.43-12.7)	1.92 (0.71-4.19)	3.49 (1.8-6.1)
Hemorrhagic stroke	18 - 34	5.78 (4.44-7.4)	4.47 (3.51-5.62)	29.49 (27.59-31.48)	51.66 (48.24-55.25)	NA	NA	13.15 (12.4-13.93)	24.63 (23.32-25.99)	14.22 (12.76-15.8)	24.64 (22.77-26.62)	7.6 (5.32-10.52)	8.58 (6.13-11.69)
Hemorrhagic stroke	35 - 54	22.44 (20.5-24.51)	25.1 (23.27-27.03)	94.22 (90.38-98.19)	160.22 (153.46-167.2)	NA	NA	24.08 (23.21-24.98)	43.06 (41.63-44.52)	43.04 (40.93-45.23)	50.4 (48.17-52.71)	34.32 (30.55-38.43)	43.8 (39.56-48.36)

Hemorrhagic stroke	55 - 64	54.75 (49.41-60.51)	65.99 (61.08-71.19)	198.3 (190.09-206.76)	329.06 (316.84-341.64)	NA	NA	46.43 (44.84-48.06)	80.17 (77.76-82.64)	118.95 (114.3-123.73)	156.95 (151.57-162.47)	60.77 (52.86-69.52)	94.35 (84.46-105.08)
Hemorrhagic stroke	65 - 74	99.74 (86.2-114.81)	105.05 (92.8-118.46)	197.45 (187.53-207.75)	302.89 (286.63-319.83)	170.07 (160.12-180.48)	251.23 (238.24-264.74)	74.31 (71.9-76.78)	119.09 (115.63-122.64)	214.24 (209.27-219.3)	292.7 (286.17-299.35)	100.31 (89.44-112.14)	162.37 (148.05-177.7)
Hemorrhagic stroke	75 - 84	225.73 (108.25-415.13)	196.6 (98.14-351.78)	305.89 (289.58-322.87)	368.64 (340.97-397.96)	413.01 (395.04-431.58)	549.93 (526.99-573.6)	163.33 (158.48-168.29)	221.33 (214.88-227.93)	441.79 (433.25-450.46)	563.96 (552.94-575.15)	180.93 (163.94-199.2)	269.93 (247.05-294.35)
Hemorrhagic stroke	85 - 114	NA	NA	446.1 (416.17-477.61)	541.05 (476.55-611.84)	777.27 (743.27-812.42)	1048.91 (998.28-1101.45)	316.2 (306.58-326.05)	372.67 (359.18-386.54)	757.98 (742.01-774.21)	987.9 (963.96-1012.29)	203.53 (180.78-228.36)	289.42 (253.15-329.44)
Pulmonary embolism	1 - 5	1.7 (0.62-3.7)	NA	1.05 (0.57-1.76)	1.28 (0.76-2.02)	NA	NA	8.67 (7.12-10.46)	7.1 (5.77-8.65)	NA	NA	NA	NA
Pulmonary embolism	6 - 17	1.3 (0.71-2.17)	1.67 (1-2.6)	4.75 (4.09-5.48)	3.28 (2.75-3.87)	NA	NA	9.8 (8.87-10.81)	7.41 (6.61-8.29)	2.78 (2.02-3.74)	2.12 (1.48-2.95)	1.92 (0.71-4.19)	1.75 (0.64-3.8)
Pulmonary embolism	18 - 34	9.91 (8.13-11.97)	4.35 (3.41-5.48)	140.84 (136.66-145.12)	84.82 (80.42-89.39)	NA	NA	72.04 (70.27-73.85)	50.26 (48.39-52.19)	60.54 (57.48-63.73)	30.89 (28.79-33.1)	15.62 (12.27-19.61)	9.87 (7.23-13.17)
Pulmonary embolism	35 - 54	20.85 (18.98-22.85)	17.46 (15.94-19.08)	378.55 (370.79-386.44)	387.63 (377.06-398.41)	NA	NA	143.49 (141.34-145.66)	161.7 (158.92-164.52)	170.74 (166.51-175.05)	135.96 (132.28-139.73)	33.06 (29.36-37.1)	43.46 (39.24-48.01)
Pulmonary embolism	55 - 64	33.07 (28.95-37.61)	42.43 (38.51-46.65)	591.53 (577.27-606.05)	618.81 (601.97-635.99)	NA	NA	198.96 (195.64-202.31)	290.69 (286.08-295.36)	335.15 (327.31-343.12)	378.35 (369.97-386.88)	76.26 (67.37-86)	128.6 (117.01-141.04)
Pulmonary embolism	65 - 74	66.82 (55.83-79.35)	68.7 (58.87-79.7)	598.16 (580.77-615.93)	606.75 (583.62-630.56)	495.56 (478.44-513.13)	603.63 (583.37-624.42)	302.59 (297.7-307.54)	371.69 (365.55-377.91)	494.42 (486.85-502.09)	558.53 (549.47-567.69)	166.01 (151.93-181.05)	213.45 (196.98-230.93)
Pulmonary embolism	75 - 84	112.89 (36.66-263.45)	89.25 (28.98-208.29)	722.56 (697.33-748.47)	721.44 (682.45-762.07)	823.36 (797.86-849.47)	869.45 (840.5-899.14)	466.88 (458.64-475.23)	476.15 (466.65-485.79)	747.48 (736.34-758.75)	768.4 (755.51-781.45)	301.73 (279.65-325.09)	283.92 (260.43-308.96)
Pulmonary embolism	85 - 114	NA	NA	812.12 (771.48-854.34)	695.84 (622.34-775.62)	993.06 (954.53-1032.75)	1034.89 (984.56-1087.12)	529.71 (517.22-542.43)	496.24 (480.64-512.22)	884.62 (867.34-902.15)	901.75 (878.87-925.07)	362.18 (331.57-394.86)	301.11 (264.07-341.89)
Appendicitis	1 - 5	17.29 (13.23-22.21)	19.31 (15.11-24.31)	37.73 (34.51-41.17)	46.74 (43.23-50.45)	NA	NA	27.05 (24.26-30.09)	37.32 (34.17-40.69)	36.57 (31.08-42.76)	47.43 (41.3-54.21)	67.86 (52.14-86.82)	69.2 (53.95-87.43)
Appendicitis	6 - 17	145.56 (138.45-152.94)	204.71 (196.48-213.19)	161.15 (157.18-165.18)	213.89 (209.48-218.38)	NA	NA	151.5 (147.74-155.33)	213.73 (209.32-218.21)	165.61 (159.32-172.08)	212.04 (205.06-219.19)	184.4 (169.62-200.12)	267.27 (250.24-285.15)
Appendicitis	18 - 34	197.18 (188.92-205.71)	185.51 (179-192.2)	175.01 (170.34-179.77)	162.96 (156.84-169.26)	NA	NA	147.45 (144.91-150.03)	208.07 (204.24-211.95)	193.93 (188.41-199.56)	206.71 (201.22-212.31)	142.04 (131.5-153.2)	159.68 (148.4-171.59)

Appendicitis	35 - 54	122.08 (117.5-126.8)	126.94 (122.79-131.19)	142.83 (138.08-147.7)	117.08 (111.31-123.07)	NA	NA	111.59 (109.7-113.51)	124.76 (122.32-127.24)	165.51 (161.34-169.75)	152.27 (148.37-156.25)	84.36 (78.38-90.68)	91.12 (84.96-97.6)
Appendicitis	55 - 64	93.43 (86.41-100.87)	105.22 (98.99-111.74)	103.02 (97.13-109.17)	101.42 (94.69-108.5)	NA	NA	83.95 (81.81-86.14)	83.61 (81.15-86.13)	147.38 (142.21-152.7)	128.35 (123.48-133.35)	68.23 (59.84-77.47)	64.13 (56.02-73.08)
Appendicitis	65 - 74	75.57 (63.85-88.83)	93.2 (81.69-105.88)	67.09 (61.37-73.2)	69.89 (62.2-78.26)	111.8 (103.76-120.3)	106.69 (98.29-115.62)	59.62 (57.46-61.83)	65.95 (63.38-68.6)	106.66 (103.16-110.25)	111.12 (107.11-115.25)	43.49 (36.44-51.51)	66.03 (57.02-76.06)
Appendicitis	75 - 84	NA	89.25 (28.98-208.29)	48.96 (42.58-56.03)	63.93 (52.74-76.8)	99.84 (91.11-109.17)	112.59 (102.36-123.56)	47.8 (45.2-50.52)	55.89 (52.67-59.25)	89.8 (85.98-93.75)	103.6 (98.92-108.45)	41.83 (33.88-51.08)	46.87 (37.64-57.68)
Appendicitis	85 - 114	NA	NA	37.76 (29.44-47.71)	48.85 (30.97-73.3)	67.77 (58.02-78.69)	114.75 (98.47-132.96)	33.24 (30.17-36.53)	43.95 (39.41-48.88)	68.21 (63.49-73.19)	79.21 (72.55-86.31)	36.47 (27.24-47.83)	47.98 (33.95-65.85)
Bells palsy	1 - 5	11.62 (8.34-15.77)	16.36 (12.51-21.01)	21.63 (19.21-24.27)	17.47 (15.35-19.79)	NA	NA	13.61 (11.64-15.8)	15.36 (13.36-17.58)	14.91 (11.48-19.03)	12.35 (9.33-16.04)	16.15 (9.04-26.64)	25.69 (16.78-37.65)
Bells palsy	6 - 17	18.04 (15.6-20.76)	15.09 (12.92-17.52)	39.49 (37.54-41.51)	27.88 (26.3-29.52)	NA	NA	27.5 (25.91-29.15)	22.89 (21.46-24.38)	20.69 (18.51-23.06)	19.53 (17.45-21.78)	44.59 (37.48-52.64)	31.73 (26.05-38.27)
Bells palsy	18 - 34	34.7 (31.29-38.38)	37.49 (34.59-40.56)	80.9 (77.74-84.16)	52.19 (48.76-55.81)	NA	NA	54.29 (52.76-55.86)	50.92 (49.04-52.86)	61.02 (57.95-64.22)	45.55 (42.99-48.21)	59.96 (53.19-67.35)	48.72 (42.59-55.48)
Bells palsy	35 - 54	60.19 (56.99-63.52)	69.81 (66.74-72.98)	139.63 (134.94-144.45)	121.17 (115.31-127.26)	NA	NA	79.32 (77.73-80.94)	94.33 (92.21-96.48)	111.38 (107.98-114.87)	103.77 (100.55-107.06)	77 (71.29-83.04)	82.78 (76.92-88.97)
Bells palsy	55 - 64	84.97 (78.29-92.08)	92.1 (86.28-98.21)	157.6 (150.3-165.16)	137.11 (129.26-145.3)	NA	NA	87.38 (85.19-89.61)	105.59 (102.82-108.42)	156.18 (150.86-161.65)	154.2 (148.87-159.67)	111.51 (100.7-123.16)	126.83 (115.32-139.18)
Bells palsy	65 - 74	102.29 (88.57-117.53)	106.59 (94.25-120.09)	127.94 (119.98-136.28)	130.45 (119.87-141.72)	169.53 (159.6-179.92)	189.87 (178.6-201.66)	93.57 (90.86-96.33)	100.14 (96.97-103.4)	185.46 (180.84-190.17)	178.76 (173.66-183.96)	131.14 (118.67-144.58)	182.3 (167.11-198.5)
Bells palsy	75 - 84	180.55 (77.95-355.75)	NA	136.25 (125.45-147.73)	122.25 (106.56-139.61)	180.36 (168.57-192.76)	190.66 (177.28-204.79)	97.06 (93.33-100.9)	103.08 (98.7-107.61)	208.91 (203.05-214.89)	195.68 (189.22-202.31)	197.02 (179.27-216.05)	232.38 (211.2-255.12)
Bells palsy	85 - 114	NA	NA	124.65 (109.09-141.8)	93.46 (67.91-125.46)	180.1 (163.96-197.39)	189.3 (168.21-212.31)	92.84 (87.66-98.24)	95.54 (88.78-102.68)	192.43 (184.44-200.67)	184.89 (174.65-195.59)	213.32 (190.02-238.7)	238.73 (205.91-275.3)
Anaphylaxis	1 - 5	NA	NA	47.6 (43.97-51.45)	71.36 (67.02-75.91)	NA	NA	46.78 (43.08-50.72)	74.56 (70.08-79.26)	77.55 (69.45-86.35)	127.49 (117.3-138.32)	12.92 (6.68-22.57)	36.56 (25.74-50.39)
Anaphylaxis	6 - 17	NA	NA	59.03 (56.64-61.49)	60.1 (57.77-62.5)	NA	NA	64.5 (62.05-67.01)	77.65 (75-80.37)	86.06 (81.55-90.76)	104.17 (99.31-109.22)	14.75 (10.8-19.68)	21.83 (17.17-27.36)

Anaphylaxis	18 - 34	NA	NA	58.15 (55.47-60.92)	35.62 (32.79-38.63)	NA	NA	55.82 (54.26-57.41)	43.54 (41.8-45.33)	79.28 (75.77-82.91)	44.24 (41.72-46.86)	10.98 (8.2-14.39)	9.44 (6.86-12.67)
Anaphylaxis	35 - 54	NA	NA	76.59 (73.13-80.18)	49.05 (45.34-52.97)	NA	NA	53.75 (52.44-55.08)	38.81 (37.45-40.2)	83.16 (80.22-86.18)	40.56 (38.56-42.64)	12.92 (10.65-15.54)	10.75 (8.71-13.13)
Anaphylaxis	55 - 64	NA	NA	55.65 (51.35-60.21)	42.55 (38.23-47.22)	NA	NA	43.02 (41.49-44.59)	34.68 (33.1-36.31)	70.99 (67.41-74.71)	45.27 (42.4-48.27)	16.33 (12.37-21.16)	13.39 (9.84-17.8)
Anaphylaxis	65 - 74	NA	NA	33.06 (29.08-37.43)	22.12 (17.89-27.04)	61.77 (55.84-68.16)	48.54 (42.94-54.68)	35.51 (33.85-37.23)	26.47 (24.86-28.17)	49.34 (46.98-51.8)	39.23 (36.86-41.71)	10.71 (7.37-15.04)	13.75 (9.82-18.72)
Anaphylaxis	75 - 84	NA	NA	20.64 (16.58-25.4)	17.38 (11.81-24.66)	48.11 (42.12-54.71)	30.4 (25.2-36.35)	21.74 (20-23.6)	22.13 (20.12-24.28)	35.87 (33.46-38.39)	31.19 (28.65-33.9)	8.71 (5.32-13.45)	13.69 (8.94-20.05)
Anaphylaxis	85 - 114	NA	NA	15.64 (10.47-22.46)	12.74 (4.67-27.73)	19.69 (14.61-25.96)	30.44 (22.37-40.48)	12.21 (10.38-14.27)	8.14 (6.26-10.42)	24.22 (21.44-27.26)	16.86 (13.87-20.31)	4.21 (1.54-9.16)	NA
Immune thrombocytopenia	1 - 5	12.19 (8.82-16.42)	16.09 (12.28-20.71)	10.93 (9.23-12.85)	14.91 (12.96-17.07)	NA	NA	11.78 (9.96-13.83)	15.94 (13.91-18.2)	11.88 (8.84-15.62)	16.32 (12.82-20.49)	14 (7.45-23.93)	23.72 (15.2-35.29)
Immune thrombocytopenia	6 - 17	6.66 (5.21-8.39)	3.95 (2.88-5.28)	9.16 (8.23-10.16)	8.27 (7.42-9.19)	NA	NA	13 (11.91-14.15)	12.39 (11.34-13.5)	9.62 (8.15-11.28)	9.28 (7.87-10.87)	5.77 (3.42-9.12)	8.15 (5.42-11.78)
Immune thrombocytopenia	18 - 34	9 (7.3-10.96)	3.08 (2.3-4.05)	35.89 (33.79-38.08)	18.57 (16.55-20.78)	NA	NA	28.99 (27.87-30.14)	16.26 (15.2-17.37)	30.72 (28.55-33.01)	12.09 (10.79-13.5)	10.34 (7.65-13.67)	6.44 (4.34-9.19)
Immune thrombocytopenia	35 - 54	11.61 (10.23-13.12)	5.41 (4.58-6.35)	47.24 (44.53-50.08)	43.54 (40.05-47.25)	NA	NA	32.07 (31.06-33.11)	25.13 (24.04-26.26)	42.74 (40.64-44.92)	24.66 (23.1-26.29)	9.26 (7.36-11.52)	4.93 (3.58-6.61)
Immune thrombocytopenia	55 - 64	14.68 (11.98-17.81)	10.78 (8.85-13)	68.2 (63.43-73.24)	66.98 (61.54-72.78)	NA	NA	40.78 (39.28-42.31)	43.01 (41.25-44.83)	59.62 (56.35-63.04)	59.49 (56.2-62.93)	10.32 (7.22-14.28)	5.98 (3.7-9.14)
Immune thrombocytopenia	65 - 74	21.07 (15.12-28.58)	15.79 (11.28-21.5)	73.07 (67.09-79.44)	81.77 (73.44-90.79)	88.98 (81.83-96.6)	114.76 (106.04-124.01)	51.23 (49.23-53.29)	64.39 (61.85-67)	80.35 (77.32-83.48)	101.28 (97.45-105.22)	14.92 (10.93-19.91)	15.47 (11.28-20.7)
Immune thrombocytopenia	75 - 84	NA	NA	80.54 (72.29-89.48)	91.43 (77.93-106.59)	114.17 (104.82-124.12)	189.55 (176.2-203.65)	63.41 (60.4-66.52)	94.18 (89.99-98.52)	100.42 (96.37-104.59)	146.68 (141.09-152.43)	22.65 (16.92-29.7)	27.91 (20.9-36.5)
Immune thrombocytopenia	85 - 114	NA	NA	78.25 (66.03-92.07)	78.61 (55.35-108.36)	122.2 (108.98-136.59)	220.67 (197.84-245.42)	72.06 (67.51-76.84)	109.95 (102.68-117.6)	107.72 (101.77-113.94)	174.36 (164.41-184.76)	27.35 (19.45-37.39)	32.82 (21.44-48.09)
Myocarditis pericarditis	1 - 5	5.1 (3.02-8.06)	5.9 (3.7-8.93)	5.76 (4.55-7.2)	7.53 (6.16-9.1)	NA	NA	4.54 (3.43-5.88)	5.22 (4.08-6.57)	5.82 (3.77-8.59)	4.63 (2.87-7.08)	NA	NA

Myocarditis pericarditis	6 - 17	5.64 (4.32-7.25)	7.81 (6.27-9.61)	8.13 (7.26-9.08)	11.6 (10.59-12.67)	NA	NA	6.39 (5.64-7.21)	9.92 (8.99-10.93)	5.89 (4.75-7.21)	10.19 (8.7-11.85)	4.49 (2.45-7.53)	11.64 (8.32-15.85)
Myocarditis pericarditis	18 - 34	4.04 (2.93-5.42)	7.86 (6.57-9.33)	28.31 (26.45-30.26)	55.44 (51.89-59.16)	NA	NA	22.89 (21.89-23.91)	52.8 (50.88-54.77)	20.88 (19.09-22.78)	37.8 (35.48-40.24)	14.35 (11.15-18.2)	61.83 (54.9-69.4)
Myocarditis pericarditis	35 - 54	5.83 (4.86-6.93)	8.78 (7.72-9.95)	56.55 (53.58-59.65)	74.76 (70.17-79.57)	NA	NA	33.15 (32.12-34.2)	50.33 (48.78-51.91)	40.2 (38.16-42.32)	48.19 (46.01-50.45)	20.82 (17.9-24.07)	50.3 (45.76-55.18)
Myocarditis pericarditis	55 - 64	6.56 (4.8-8.74)	12.06 (10.02-14.41)	66.22 (61.52-71.19)	84.92 (78.77-91.42)	NA	NA	41.99 (40.48-43.55)	60.21 (58.12-62.35)	67.04 (63.56-70.65)	84.74 (80.8-88.82)	37.83 (31.65-44.86)	65.84 (57.62-74.9)
Myocarditis pericarditis	65 - 74	11.82 (7.49-17.73)	17.36 (12.62-23.31)	62.31 (56.79-68.21)	73.62 (65.72-82.2)	81.48 (74.64-88.78)	115.12 (106.38-124.38)	46.16 (44.27-48.12)	66.25 (63.67-68.9)	76.4 (73.45-79.45)	100.47 (96.66-104.4)	49.33 (41.8-57.83)	85.64 (75.33-96.96)
Myocarditis pericarditis	75 - 84	NA	NA	55.47 (48.66-62.96)	67.3 (55.79-80.47)	100.25 (91.51-109.6)	149.65 (137.81-162.22)	51.42 (48.71-54.23)	72.42 (68.76-76.24)	91.23 (87.38-95.21)	117.96 (112.95-123.13)	61.88 (52.12-72.93)	101.17 (87.36-116.53)
Myocarditis pericarditis	85 - 114	NA	NA	61.51 (50.74-73.89)	57.35 (37.8-83.45)	116.27 (103.38-130.32)	133.59 (115.97-153.13)	46.38 (42.74-50.24)	52.49 (47.51-57.85)	96.61 (90.97-102.5)	122.28 (113.97-131.03)	42.78 (32.73-54.96)	80.83 (62.25-103.22)
Disseminated intravascular coagulation	1 - 5	24.67 (19.76-30.43)	31.65 (26.19-37.9)	2.69 (1.89-3.73)	3.91 (2.94-5.08)	NA	NA	1.75 (1.1-2.65)	1.45 (0.89-2.24)	1.63 (0.66-3.36)	1.32 (0.49-2.88)	NA	NA
Disseminated intravascular coagulation	6 - 17	17.58 (15.17-20.27)	18.25 (15.85-20.91)	2.92 (2.41-3.51)	2.87 (2.38-3.43)	NA	NA	1.23 (0.91-1.62)	1.53 (1.18-1.95)	1.58 (1.02-2.34)	1.94 (1.33-2.74)	NA	NA
Disseminated intravascular coagulation	18 - 34	61.91 (57.32-66.76)	17.23 (15.29-19.35)	14.92 (13.58-16.36)	12.91 (11.23-14.77)	NA	NA	5.34 (4.86-5.85)	4.43 (3.89-5.03)	6.12 (5.17-7.19)	3.76 (3.05-4.58)	NA	1.5 (0.6-3.09)
Disseminated intravascular coagulation	35 - 54	54.33 (51.29-57.5)	31.23 (29.19-33.37)	24.32 (22.39-26.38)	35.51 (32.36-38.87)	NA	NA	5.93 (5.5-6.38)	7 (6.43-7.6)	9.54 (8.56-10.6)	7.09 (6.26-7.98)	2.74 (1.76-4.08)	3.47 (2.36-4.93)
Disseminated intravascular coagulation	55 - 64	64.74 (58.92-70.98)	83.41 (77.87-89.24)	46.74 (42.81-50.94)	56.26 (51.28-61.6)	NA	NA	8.44 (7.77-9.15)	12.34 (11.4-13.33)	21.21 (19.28-23.29)	22.2 (20.2-24.33)	4.3 (2.41-7.09)	6.84 (4.38-10.17)
Disseminated intravascular coagulation	65 - 74	111.58 (97.23-127.46)	167.12 (151.57-183.83)	41.3 (36.84-46.15)	58.45 (51.44-66.15)	21.22 (17.8-25.1)	32.07 (27.54-37.13)	11.85 (10.9-12.86)	15.13 (13.91-16.42)	29.65 (27.82-31.57)	41.07 (38.65-43.61)	4.54 (2.48-7.62)	7.22 (4.47-11.03)
Disseminated intravascular coagulation	75 - 84	157.98 (63.52-325.49)	250.18 (136.78-419.76)	43.85 (37.82-50.56)	56.62 (46.12-68.8)	37.33 (32.08-43.2)	48.9 (42.25-56.31)	15.09 (13.64-16.65)	18.56 (16.72-20.53)	39.55 (37.03-42.2)	53.43 (50.08-56.94)	10.45 (6.7-15.55)	11.58 (7.26-17.54)
Disseminated intravascular coagulation	85 - 114	NA	NA	39.91 (31.34-50.11)	42.47 (25.94-65.59)	43.72 (35.96-52.65)	66.74 (54.48-80.95)	14.22 (12.24-16.43)	21.33 (18.2-24.84)	35.41 (32.03-39.05)	54.25 (48.77-60.18)	3.51 (1.14-8.18)	7.57 (2.78-16.48)

Encephalomyelitis	1 - 5	8.5 (5.74-12.14)	6.97 (4.55-10.21)	5.46 (4.28-6.87)	5.61 (4.44-6.99)	NA	NA	2.86 (2.01-3.97)	3.33 (2.44-4.45)	7.92 (5.48-11.06)	4.41 (2.69-6.81)	NA	NA
Encephalomyelitis	6 - 17	3.7 (2.64-5.04)	4.39 (3.26-5.78)	5.7 (4.97-6.5)	5.09 (4.43-5.82)	NA	NA	2.83 (2.33-3.39)	3.3 (2.77-3.9)	5.89 (4.75-7.21)	5.21 (4.17-6.44)	1.6 (0.52-3.74)	2.33 (1.01-4.59)
Encephalomyelitis	18 - 34	5.97 (4.6-7.6)	3.57 (2.72-4.6)	8.99 (7.95-10.12)	10.17 (8.68-11.83)	NA	NA	3.25 (2.88-3.65)	3.9 (3.39-4.46)	4.22 (3.44-5.12)	4.68 (3.89-5.59)	4.01 (2.41-6.26)	3.43 (1.96-5.58)
Encephalomyelitis	35 - 54	3.96 (3.17-4.88)	4.09 (3.37-4.91)	19.11 (17.4-20.94)	24.9 (22.28-27.75)	NA	NA	3.65 (3.31-4.01)	4.84 (4.37-5.35)	7.75 (6.87-8.71)	7.06 (6.24-7.96)	3.55 (2.41-5.03)	4.81 (3.48-6.49)
Encephalomyelitis	55 - 64	5.56 (3.95-7.6)	7.71 (6.1-9.63)	28.28 (25.23-31.58)	29.69 (26.1-33.63)	NA	NA	5.26 (4.73-5.83)	6.92 (6.22-7.67)	15.97 (14.3-17.78)	17.27 (15.52-19.16)	4.3 (2.41-7.09)	6.84 (4.38-10.17)
Encephalomyelitis	65 - 74	4.62 (2.11-8.78)	9.47 (6.07-14.09)	24.43 (21.03-28.23)	30.73 (25.71-36.45)	14.51 (11.71-17.77)	20.78 (17.17-24.92)	5.98 (5.31-6.71)	8.32 (7.42-9.29)	20.39 (18.88-22)	25.08 (23.19-27.08)	7.14 (4.47-10.81)	9.62 (6.39-13.91)
Encephalomyelitis	75 - 84	NA	NA	19.72 (15.75-24.38)	26.35 (19.36-35.03)	19.7 (15.94-24.08)	36.73 (31-43.22)	6.96 (5.99-8.04)	10.17 (8.83-11.66)	24.79 (22.8-26.91)	30.86 (28.33-33.55)	4.79 (2.39-8.57)	6.32 (3.26-11.03)
Encephalomyelitis	85 - 114	NA	NA	22.65 (16.32-30.62)	21.23 (10.18-39.05)	29.53 (23.23-37.02)	36.28 (27.4-47.11)	6.8 (5.45-8.38)	7.11 (5.35-9.25)	23.43 (20.7-26.42)	34.34 (30.01-39.12)	4.91 (1.97-10.11)	NA
Narcolepsy	1 - 5	2.27 (0.98-4.47)	3.22 (1.66-5.62)	0.97 (0.52-1.66)	1.56 (0.98-2.36)	NA	NA	NA	NA	NA	NA	NA	NA
Narcolepsy	6 - 17	10.36 (8.53-12.47)	10.09 (8.33-12.11)	13.26 (12.15-14.46)	14.06 (12.94-15.24)	NA	NA	NA	NA	9.68 (8.21-11.34)	8.37 (7.03-9.89)	NA	NA
Narcolepsy	18 - 34	21.67 (18.99-24.62)	16.45 (14.55-18.52)	46.49 (44.1-48.97)	33.01 (30.29-35.91)	NA	NA	NA	NA	52.97 (50.11-55.95)	29.93 (27.87-32.11)	1.27 (0.46-2.76)	1.93 (0.88-3.67)
Narcolepsy	35 - 54	6.6 (5.57-7.77)	9.21 (8.12-10.41)	91.82 (88.02-95.74)	56.65 (52.66-60.86)	NA	NA	NA	NA	57.67 (55.22-60.19)	32.64 (30.85-34.51)	2.06 (1.22-3.25)	2.24 (1.37-3.46)
Narcolepsy	55 - 64	4.42 (3-6.27)	7.71 (6.1-9.63)	61.36 (56.83-66.14)	39.8 (35.62-44.32)	NA	NA	NA	NA	53.61 (50.5-56.85)	36.6 (34.02-39.31)	1.72 (0.63-3.74)	4.27 (2.39-7.05)
Narcolepsy	65 - 74	6.68 (3.56-11.42)	8.68 (5.44-13.14)	42.5 (37.97-47.43)	30.97 (25.93-36.71)	37.76 (33.15-42.83)	40.5 (35.39-46.14)	NA	NA	38.01 (35.93-40.17)	34.71 (32.49-37.05)	NA	NA
Narcolepsy	75 - 84	NA	NA	25.98 (21.4-31.27)	26.91 (19.84-35.68)	32.57 (27.67-38.08)	41.56 (35.44-48.43)	NA	NA	27.57 (25.47-29.79)	33.8 (31.14-36.62)	2.18 (0.71-5.08)	NA

Narcolepsy	85 - 114	NA	NA	20.5 (14.5-28.13)	23.36 (11.66-41.79)	25.6 (19.76-32.63)	32.39 (24.04-42.71)	NA	NA	19.55 (17.07-22.3)	28.42 (24.49-32.79)	NA	NA
Guillain-Barre syndrome	1 - 5	1.42 (0.46-3.31)	2.68 (1.29-4.93)	0.97 (0.52-1.66)	0.92 (0.49-1.58)	NA	NA	NA	0.65 (0.3-1.24)	1.86 (0.8-3.67)	1.1 (0.36-2.57)	5.38 (1.75-12.56)	NA
Guillain-Barre syndrome	6 - 17	0.74 (0.32-1.46)	1.67 (1-2.6)	0.62 (0.39-0.92)	0.88 (0.62-1.22)	NA	NA	0.57 (0.36-0.85)	0.67 (0.44-0.97)	1.08 (0.63-1.72)	1.27 (0.79-1.95)	1.92 (0.71-4.19)	2.04 (0.82-4.2)
Guillain-Barre syndrome	18 - 34	3.03 (2.09-4.25)	2.24 (1.57-3.08)	3.57 (2.94-4.31)	2.56 (1.84-3.46)	NA	NA	2.03 (1.74-2.35)	1.92 (1.57-2.33)	3.35 (2.66-4.16)	1.99 (1.49-2.62)	1.9 (0.87-3.61)	3.22 (1.8-5.31)
Guillain-Barre syndrome	35 - 54	2.05 (1.49-2.74)	4.09 (3.37-4.91)	7.84 (6.76-9.05)	6.13 (4.87-7.62)	NA	NA	2.73 (2.44-3.05)	3.25 (2.86-3.67)	4.27 (3.63-5)	3.79 (3.2-4.47)	1.94 (1.13-3.11)	4.37 (3.11-5.97)
Guillain-Barre syndrome	55 - 64	3.56 (2.31-5.26)	4.85 (3.58-6.41)	10.18 (8.39-12.23)	9.38 (7.41-11.7)	NA	NA	3.18 (2.77-3.63)	5.29 (4.68-5.95)	7.84 (6.68-9.14)	8.63 (7.41-10)	2.01 (0.81-4.13)	7.41 (4.84-10.85)
Guillain-Barre syndrome	65 - 74	3.08 (1.13-6.71)	5.92 (3.31-9.76)	7.57 (5.73-9.81)	8.15 (5.68-11.33)	9.98 (7.69-12.75)	11.29 (8.67-14.44)	4.07 (3.53-4.69)	5.69 (4.95-6.5)	8.68 (7.7-9.75)	12.46 (11.14-13.89)	6.81 (4.22-10.41)	11.69 (8.09-16.33)
Guillain-Barre syndrome	75 - 84	NA	NA	4.64 (2.83-7.16)	12.33 (7.73-18.67)	7.05 (4.88-9.85)	17.99 (14.05-22.69)	5.17 (4.34-6.12)	6.9 (5.8-8.14)	9.22 (8.02-10.54)	15.01 (13.26-16.92)	6.53 (3.66-10.78)	15.27 (10.23-21.93)
Guillain-Barre syndrome	85 - 114	NA	NA	5.93 (2.96-10.61)	NA	13 (8.95-18.25)	23.32 (16.33-32.29)	4.87 (3.74-6.23)	7.11 (5.35-9.25)	8.37 (6.77-10.23)	19.14 (15.95-22.79)	4.21 (1.54-9.16)	13.88 (6.93-24.84)
Transverse myelitis	1 - 5	NA	NA	0.67 (0.31-1.28)	1.14 (0.65-1.84)	NA	NA	0.72 (0.33-1.36)	0.43 (0.16-0.95)	1.16 (0.38-2.72)	NA	NA	NA
Transverse myelitis	6 - 17	NA	NA	1.21 (0.89-1.6)	1.05 (0.76-1.41)	NA	NA	1.2 (0.89-1.59)	1.27 (0.95-1.66)	1.01 (0.58-1.64)	0.85 (0.46-1.42)	NA	NA
Transverse myelitis	18 - 34	NA	NA	5.21 (4.44-6.09)	3.9 (3-4.98)	NA	NA	4.64 (4.2-5.11)	3.34 (2.87-3.87)	4.22 (3.44-5.12)	2.8 (2.2-3.52)	1.27 (0.46-2.76)	NA
Transverse myelitis	35 - 54	0.36 (0.16-0.72)	0.36 (0.17-0.66)	12.1 (10.75-13.57)	9.01 (7.46-10.78)	NA	NA	8.37 (7.86-8.9)	6.27 (5.73-6.84)	11.52 (10.44-12.68)	5.29 (4.59-6.08)	2.52 (1.58-3.81)	1.01 (0.46-1.91)
Transverse myelitis	55 - 64	NA	NA	10.54 (8.71-12.63)	7.81 (6.03-9.96)	NA	NA	7.2 (6.58-7.86)	7.11 (6.4-7.87)	11.5 (10.08-13.05)	8.68 (7.45-10.06)	1.43 (0.47-3.34)	1.42 (0.46-3.32)
Transverse myelitis	65 - 74	NA	NA	6.9 (5.16-9.05)	5.12 (3.21-7.75)	11.39 (8.93-14.32)	6.99 (4.97-9.55)	6.76 (6.05-7.54)	5.34 (4.63-6.13)	8.77 (7.79-9.84)	7.21 (6.22-8.32)	NA	NA
Transverse myelitis	75 - 84	NA	NA	4.87 (3.02-7.45)	4.48 (1.94-8.84)	8.5 (6.1-11.53)	8.87 (6.18-12.33)	5.17 (4.34-6.12)	4.42 (3.55-5.43)	6.66 (5.65-7.8)	6.04 (4.95-7.29)	NA	NA

Transverse myelitis	85 - 114	NA	NA	NA	NA	4.33 (2.16-7.75)	6.48 (3.11-11.91)	2.16 (1.44-3.13)	2.84 (1.78-4.3)	4.4 (3.27-5.81)	5.32 (3.7-7.4)	NA	NA

Appendix Figure 1: database-specific incidence rates and meta-analytic pooled rates with 95% prediction intervals

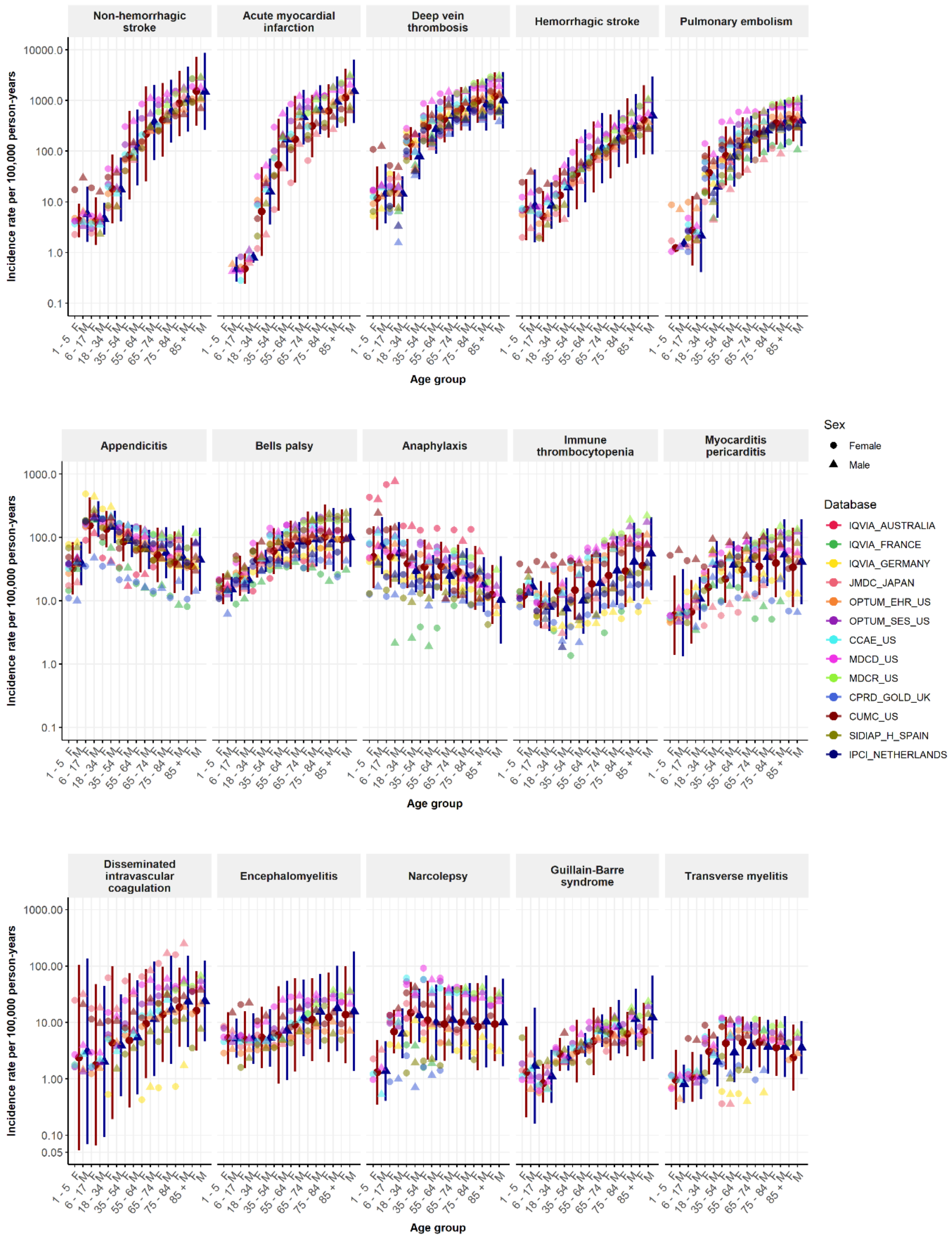

Supplement: Supplementary file 1 — Supplementary information: Appendix tables 1-4 and figure 1 [file lix065802.ww.pdf]
